# Supplementary material for: Repurposing a chemosensory macromolecular machine
Source: Nat Commun. 2020 Apr 27;11:2041. doi: 10.1038/s41467-020-15736-5 (PMC7184735; doi:10.1038/s41467-020-15736-5)
Supplement: Supplementary file 1 — Supplementary Information [file 41467_2020_15736_MOESM1_ESM.pdf]

Supplementary Information

Repurposing a chemosensory macromolecular machine

Ortega D. R. *et. al.*

Supplementary Discussion

Our results also shed light on the biological function of CheD. CheD is thought to interact with chemoreceptors in an adaptation mechanism together with CheC and CheY[1], but more recent results showed that CheD from *Bacillus subtilis* is able to deamidate chemoreceptors *in vitro* without CheC[2]. Our results provide two further pieces of evidence supporting the idea that CheD is able to perform a biological role independently of CheC. First, the ancestral F7 system included *cheD* but not *cheC*. Second, *cheD* co-evolved with the ancestral F7 *cheY* (it was lost in the same evolutionary step), pointing to a functional link.

A recent study in *Comamonas testosteroni*, an organism with a stage 4 F7 system, shows that the kinase CheA is able to phosphorylate both the ancient CheY as well as the recently acquired CheY-F6-like[3]. Deletion of the CheY-F6-like protein completely abolished chemotaxis response, while the deletion of CheY-F7 only partially affected it. The study further shows that CheY-F7 has a much faster auto-dephosphorylation rate than CheY-F6-like. The authors interpreted these results such that the CheY-F6-like is the primary response regulator, and CheY-F7 may act as a phosphate sink. These conclusions are based on previous work in organisms with multiple CheY genes per chemosensory cluster.

However, because CheY-F7 in stages 1 and 2 is the sole response regulator of the system, we hypothesize that it plays a major role in the control of a yet-unknown cellular process, at least in stages 1 and 2. The rapid auto-dephosphorylation does not necessarily imply a phosphate sink as the main biological function of this CheY. Thus, in the intermediate stages of the extant β-Proteobacteria, including the system in *C. testosteroni*, the CheY-F7 may retain both the older unknown function as well as its new role in the control of the flagellar motor.

On the other hand, the immediate loss of McpA and adjacent genes from stage 1 to 3, might indicate the loss of the original F7 function. If this were true, why would the system keep a conserved response regulator (cheY) for a lost function? One hypothesis is that the original CheY and CheD might be serving in an auxiliary feedback loop in addition to the canonical CheB/CheR adaptation mechanism in stages 3 and 4. This could compensate response time of the flagellar control as the system acquired mutations to accommodate the new components and function,

providing flexibility. In the stage 5, the system evolved to perform optimum response without the need of CheY/CheD adaptation mechanism, resulting in loss of these components in that stage.

Imaging four γ-Proteobacteria with both F6 and F7 systems by cryo-ET, we observed that the arrays formed by different chemosensory systems were both separate and structurally distinct. This is consistent with previous studies showing physical separation of the arrays from different chemosensory gene clusters in *V. cholerae* (F6 and F9 systems [4]) and *P. aeruginosa* (F6 and F7 systems[5]). It is also consistent with experiments in *E. coli* showing that engineered chemoreceptors with additional heptads cannot form arrays with shorter, native chemoreceptors, likely because of a large physical mismatch in the CheA/CheW layers[6].

In all cases, we observed that the F7 arrays in non-enteric γ-Proteobacteria were membrane-associated, but it remains unclear how this is achieved. In other arrays, N-terminal hydrophobic alpha-helices of chemoreceptors mediate membrane insertion. Aer2-like receptors in *V. cholerae*, *P. aeruginosa* and *S. oneidensis*, however, lack any predicted transmembrane regions. The *M. alcaliphilum* Aer2-like receptor has two small fragments of transmembrane regions (10 and 14 residues), but these are likely too short to attach the receptor to the membrane. One possibility is that the receptors are post-translationally modified for membrane attachment. Another possibility is that another protein serves as a membrane anchor. Our work ruled out one such candidate – the McpA receptor in the same gene cluster; *ΔmcpA* F7 arrays were still attached to the membrane.

One of the most striking features of the F7 arrays in non-enteric γ-Proteobacteria was the presence of additional density layers between the CheA/CheW baseplate and the IM. Based on our homology models of the receptors, we propose that these layers correspond to domain features (Fig. 2B). The L2 layer matched the PAS domain present in Aer2-like receptors in all four species. The fainter (possibly less-ordered) L3 layer in *V. cholerae* and *S. oneidensis* matched the additional PAS domain in the Aer2-like receptors from these species. This suggests that PAS domains might mediate intra- and inter-trimer interactions, potentially contributing to cooperativity in the signaling array. The L1 layer matched the junction between the HAMP and signaling domains, which is puzzling because this chemoreceptor region is predicted to have low molecular density[7]. It is unlikely that this density is produced by another known chemotaxis protein. For example

CheR, that binds the chemoreceptor in that area, is not expected to have enough abundance to generate a visible density layer[8,9]. Furthermore, previous cryo-ET of *in vitro* preparations containing only *E. coli* CheA, CheW and Tsr showed a similar layer in that region, suggesting one or more of these proteins alone is responsible for the L1 layer[10]. Similarly, the L3 layer in *P. aeruginosa* F7 array appears to be located between 2 HAMP domains. Interestingly, both *P. aeruginosa* L3 and the L1 layer in all organisms coincide with a coupling double alpha-helix linker between two four-helical bundles. However, the composition of these layers remains unclear.

Another mystery is the function of the F7 chemosensory array in non-enteric γ-Proteobacteria. Flagellar motility in these organisms is controlled by the F6 chemosensory system[11–13], which is expressed under a variety of conditions. In contrast, the *P. aeruginosa* and *V. cholerae* F7 system is only expressed when cells are grown in stressful conditions such as into late stationary phase, induced by the stress-related sigma factor RpoS[5,14,15]. Expression of the F7 system in different conditions has not been studied in *S. oneidensis* or *M. alcaliphilum*, but both organisms live in unique and challenging environments which may be poorly mimicked by laboratory growth; *S. oneidensis* is a facultative anaerobe adapted to changing environments [16] and *M. alcaliphilum* is a haloalkaliphilic methanotroph[17]. While we did not test different growth conditions for *M. alcaliphilum*, we did observe that formation of F7 arrays in *S. oneidensis* was dependent on culture conditions. Another clue is that both *P. aeruginosa* and *V. cholerae* are capable of sensing oxygen, which binds to the PAS-heme domains of Aer2 receptors to activate signaling[18,19]. We therefore favor the working model that the older F7 systems are part of an emergency response system activated by stress conditions, perhaps related to the availability of oxygen. The McpA receptor may also be an important mediator of this response. McpA has no sensory domain, but has been implicated in taxis toward trichloroethylene[20]. A previous study in *P. aeruginosa* showed that McpA physically co-localizes with F6 system proteins[5]. Here we find that despite being part of the F6 system, McpA co-evolved with the F7 system, suggesting that McpA may bridge the two systems to provide additional inputs to the flagellar control system in response to stress.

Supplementary Figures

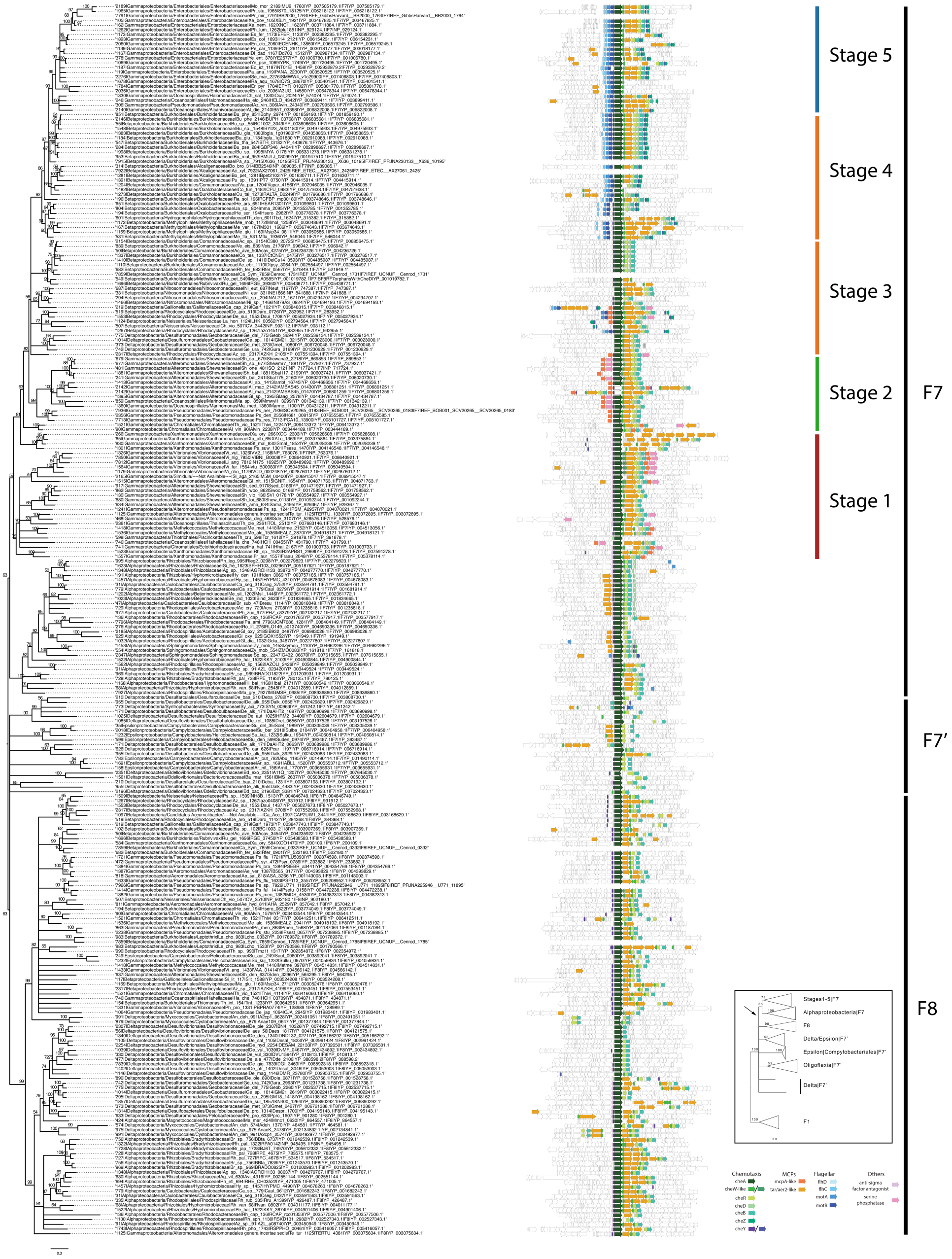

Supplementary Figure 1: Phylogeny of CheA, CheB and CheR concatenated alignments of F7 and F8 systems and gene neighborhood of 15 genes up and downstream from CheA. The inlet shows a CheABR tree with additional sequences from F1 systems. The tree is rooted by the common ancestor between F1 systems and the F7/F8 systems.

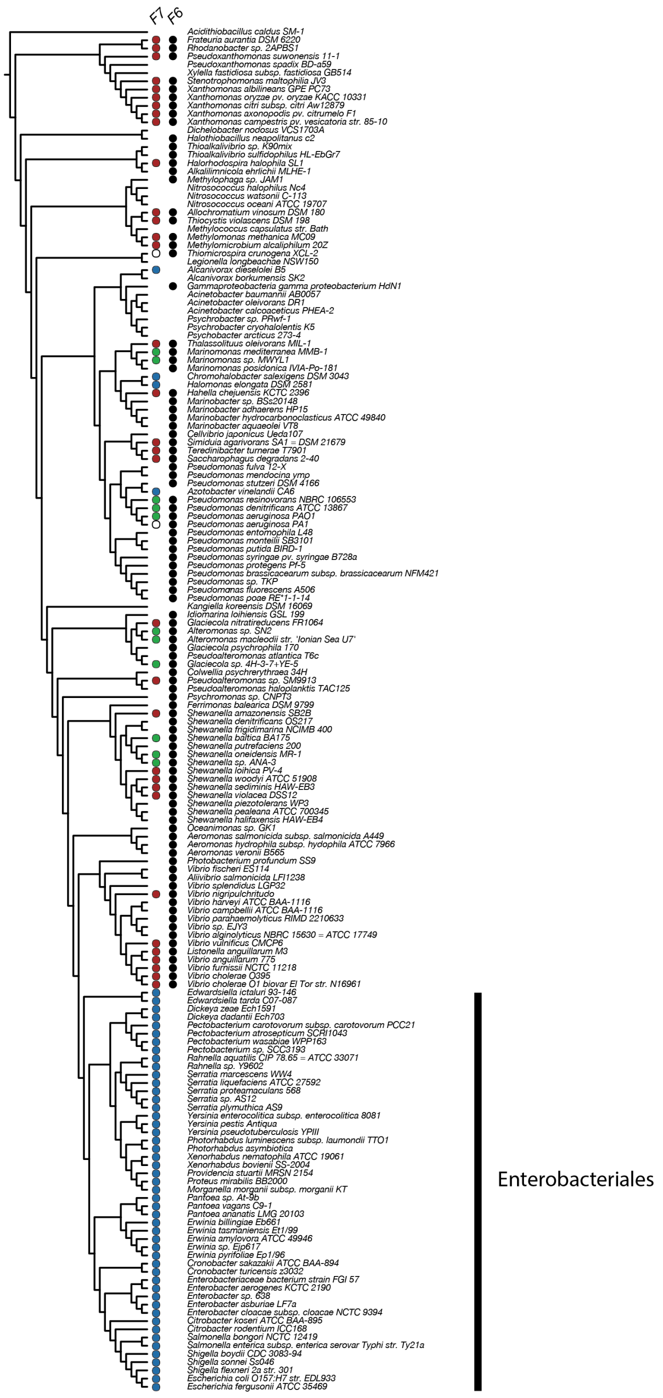

Supplementary Figure 2: Phylogenetic profile of the F7 and F6 systems in g-Proteobacteria shows that only organisms with F7 stage 1 (red) or stage 2 (green) systems also has F6 systems. Organisms with F7 stage 5 (blue) does not have F6 systems. Note that the distribution of stage 1and stage 2 are mixed in the non-enteric group. Genomes with empty circles were genomes included in this part of the research but not in the analysis of classifying the F7 systems.

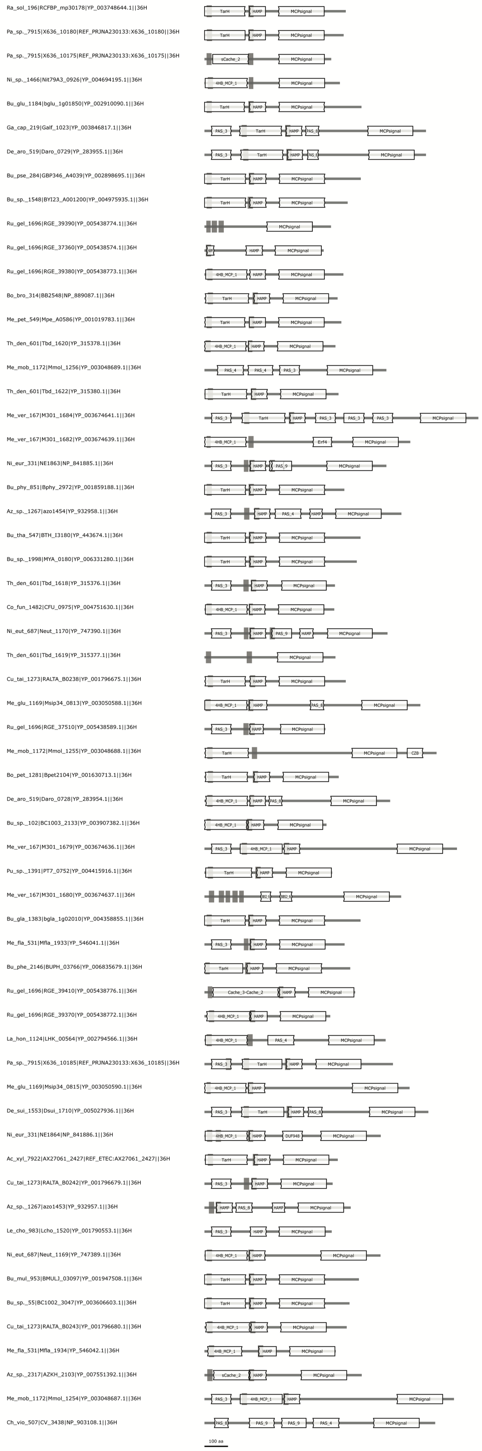

Supplementary Figure 3: Protein domain architecture of chemoreceptors in the gene cluster of stage 3 and 4 of F7 systems shows the high incidence of receptors with transmembrane regions and periplasmic sensory domains (TarH, Cache superfamily, 4HB_MCP_1).

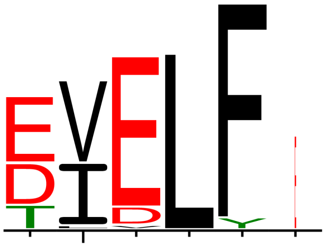

Supplementary Figure 4: Sequence logo of the C-terminal of McpA-like sequences.

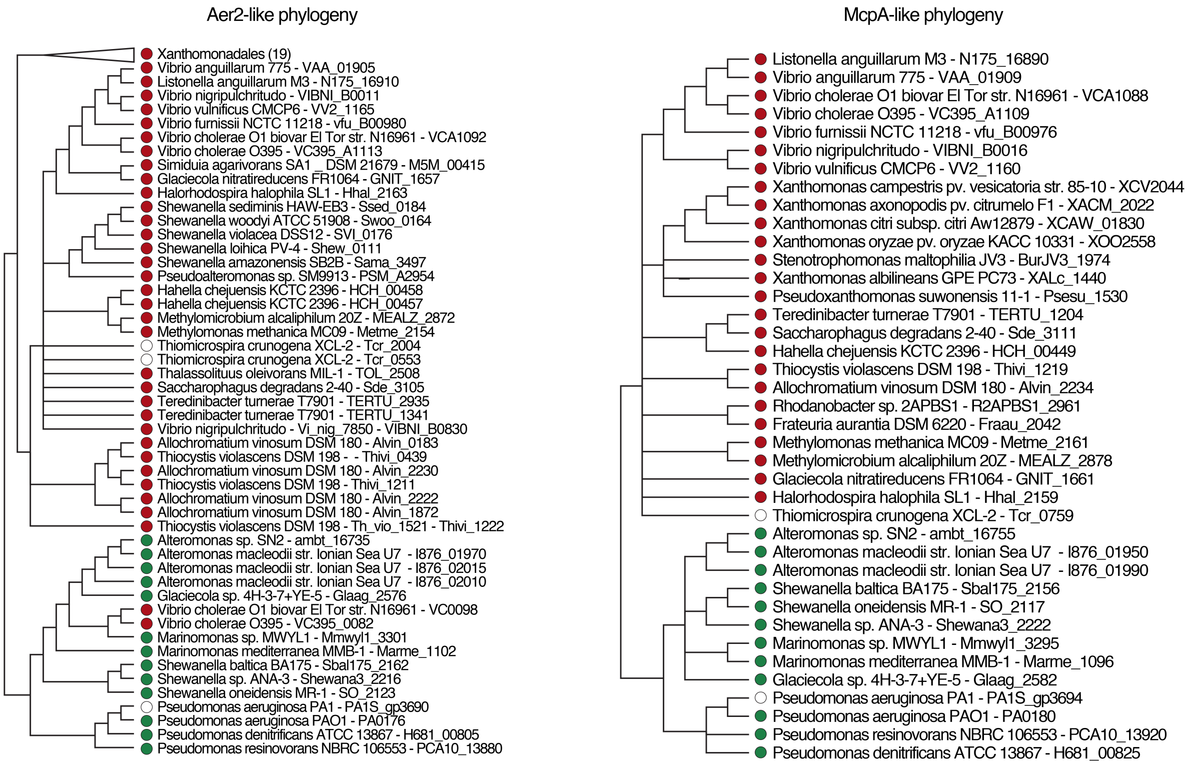

Supplementary Figure 5: Phylogenetic tree of Aer2-like receptors and McpA-like receptors. The tags in the tips are built using the name of the organism and the locus of the receptor. Tips with red dots belong to chemoreceptors from stage 1 and green to stage 2. The only exception to the monophyletic distribution of Aer2-like receptors were in *V. cholerae*, where an addition to the Aer2 homolog, a second, orphan 36H receptor (VC0098) was likely introduced by a recent lateral gene transfer from *Marinomonas*. Genomes with empty circles were genomes included in this part of the research but not in the analysis of classifying the F7 systems.

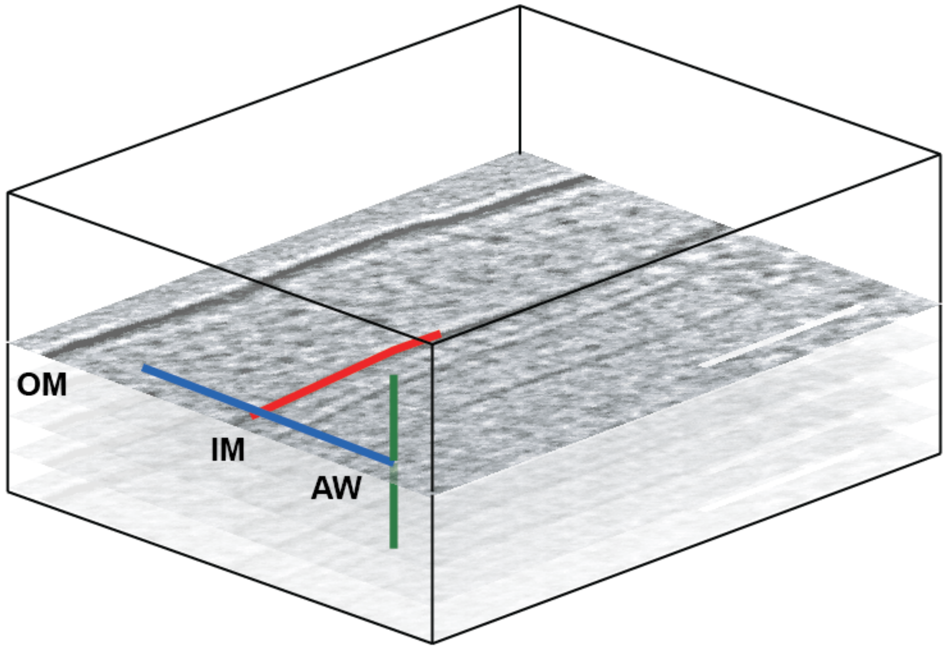

Supplementary Figure 6: The 1D electron density profile is a collapse of a 3D sub-volume. For each model point (red), the algorithm extends a profile perpendicular to the model points (blue). Then, it averages the intensity of the pixels perpendicular to the profile in the slices above and below (green). The final 1D profile is an average of the profiles calculated for each pixel of the model point.

Supplementary Tables

Supplementary Table 1: Presence and absence of the chemosensory arrays in imaged strains of *V. cholerae*, *P. aeruginosa*, *S. oneidensis*, and *M. alcaliphilum*.

	**Imaged cell poles**	**Short array**	**Tall array**
Vibrio cholerae			
Wild-type C6706	29	20	7
Δmcp (VCA1088)	20	19	5
ΔF7 cheW, cheW, cheA (VCA1093, VCA1094, VCA1095)	29	18	0
Δmcp (Aer2/VCA1092)	27	24	0
Pseudomonas aeruginosa			
Wild -type PAO1	16	7	5
ΔF6 cheW	15	8	6
Δmcp 'mcpA' (PAO180)	8	4	2
ΔF7 cheW (PAO177)	33	12	0
ΔF7 cheA (PAO 178)	34	24	0
Δmcp 'aer2', 'mcpB' (PAO176)	21	11	0
Shewanella oneidensis MR-1			
Chemostat growth	29	20	7
Batch culture growth	29	18	0
Methylomicrobium alcaliphilum 20Z			
Wild-type	8	5	2

Supplementary Table 2: Chemosensory gene clusters in the genomes of *V. cholerae*, *P. aeruginosa*, *S. oneidensis* and *M. alcaliphilum*.

	**Classification**	**Alternative name in literature**	**Function**	**Gene cluster**
Vibrio cholerae				
Cluster I	F9	-	Unknown	VC1394-VC1405
Cluster II	F6	-	Chemotaxis	VC2059-VC2064
Cluster III	F7	-	Unknown	VCA1090-VCA1095
Pseudomonas aeruginosa				
Cluster I/V	F6	Che I	Chemotaxis	PA1457-PA1464
Cluster II	F7	Che II	Unknown	PA0173-PA0180
Cluster III	ACF	Wsp	Biofilm formation	PA3703-PA3708
Cluster IV	TFP	Chp	Twitching motility	PA0410-PA0415
Shewanella oneidensis				
Cluster I	F7	CheA-1	Unknown	SO_2117-SO_2126
Cluster II	F6	CheA-3	Chemotaxis	SO_3200-SO_3209
Methylomicrobium alcaliphilum				
Cluster I	F7	-	Unknown	MEALZ_2869-MEALZ_2879
Cluster II	F8	-	Unknown	MEALZ_2939 - MEALZ_2942
Cluster III	F6	-	Unknown	MEALZ_3148 - MEALZ_3158

Supplementary Table 3: Number of cells and sub-tomograms used to generate averages shown in Figure 2.

	Cells (tomograms)	Sub-tomograms	Pixel size (nm)
P. aeruginosa	5	1113	0.64
V. cholarae	6	265	1.3
S. oneidensis	5	327	1
M. alcaliphilum	2	1448	1

Supplementary Table 4: Locations of electron density layers in arrays. Distances are measured from the CheA/CheW baseplate in nanometers. Uncertainties reported are the expanded standard uncertainty.

Layers (F7)	*V. cholerae*	*P. aeruginosa*	*S. oneidensis*	*M. alcaliphilum*
Inner membrane (IM)	38.4±1.9 nm	40.3±1.8 nm	35.5±2.7 nm	35.1±2.8 nm
Layer 3 (L3)	29.5 ±1.9 nm	30.7±1.8 nm	30.7±4.3 nm	-
Layer 2 (L2)	24.8±1.9 nm	24.0±1.8 nm	24.0±2.7 nm	25.3±4.4 nm
Layer 1 (L1)	17.5±1.9 nm	17.9±2.9 nm	17.3±2.7 nm	17.4±4.4 nm
Signaling Layer (SL)	-	7.0±1.8 nm	6.7±4.3 nm	7.5±2.8 nm
**Layers (F6)**				
Inner membrane (IM)	25.8±1.9 nm	24.5±2.7 nm	24.3±1.8 nm	25.6±5.6 nm

Supplementary Table 5: *P. aeruginosa* strains

Strain name	PA ORF	Gene
PW1307	PA0178	CheA F7 system
PW1305	PA0177	CheW F7 system
PW1312	PA0180	MCPA
PW3654	PA1464	CheW F6 system

Supplementary Table 6: Cryo-Electron Tomograms used in this study available on ETDB. Tomograms can be found on ETDB by the Open Index Protocol id (OIP id).

Jensen Lab id	OIP id	Organism
ab2015-06-02-1	6cef7c25e66ce42e4e2440490a68845a551303 77f7bc9e096c48fd83b47ae9cd	*Vibrio cholerae*
ab2015-06-02-2	98d93b8c8e390eb6c200fc2d30c9626cb1a7c1 40cd930a60eddb3a179aa8226b	*Vibrio cholerae*

Jensen Lab id	OIP id	Organism
ab2015-06-02-3	39a5d8490ee3823eaf785268aba1593d3be05b<br>8bf6cc029d2e0c247bb7b0a6ba	*Vibrio cholerae*
ab2015-06-02-5	2620f1ee27c5e63853bb3f3d09796b19d7bd91<br>fbdb5781daa892d68fb31be735	*Vibrio cholerae*
ab2015-06-02-6	9ba344e00d31084d2aef8a799f3089e46ead3b<br>28a02c0e73c5852337f67121fc	*Vibrio cholerae*
ab2015-06-02-7	1e00525c41a9faaa849befd686b70757a6f25a9<br>ceacbcde951b6eeef99006f09	*Vibrio cholerae*
ab2015-06-02-8	3cb8dc276fb373a99af2ee54301fcb0989eb003<br>7b9c3c2bd21f8e035f0f98215	*Vibrio cholerae*
ab2015-06-02-9	5739b1525b6a0e906de41a8e024e0b502f964<br>e19193feed1a62ced0e41b5dc17	*Vibrio cholerae*
ab2015-06-02-10	7e869e97212b3df06af70decf577ef3836c1ec4<br>787f1cbe677da60aca3bda727	*Vibrio cholerae*
ab2015-06-02-11	8329b3a0b5373c89d5280c42ca8aabb19a38a6<br>ee03e5910275b82776ff8745fa	*Vibrio cholerae*
ab2015-06-02-12	de04da0f550b2f37d4a0847e13b637877ed699<br>4bb5d8d0c24c87ad9f3cf52924	*Vibrio cholerae*
ab2015-06-02-13	4caf0202a48c92372ca3a24101d9c5e2b446fad<br>bf80109b5d0710598a11fa3aa	*Vibrio cholerae*
ab2015-06-02-14	b2f7beb0de38a1eb8d0eab4d8493616c912293<br>1ec0490e1bb5b45474a032098f	*Vibrio cholerae*
ab2015-06-02-15	bd8198a02bd66929811014f1b15a243f47054<br>33876cb56c784bdc8fb91378c00	*Vibrio cholerae*
ab2015-06-02-16	87c298d70069315af599618c344d80bf2fc48f4<br>4c0057002798241842b58b5c9	*Vibrio cholerae*
ab2015-06-02-17	066cf7fae820b463b2e7405a9078e97b6de991<br>0587458dfd904759d75fb4ed2c	*Vibrio cholerae*
ab2015-06-02-18	82fdc6778793542fad9bfe7c18a6fa972dc3bf23<br>0b2e108aa53a5807811bdad7	*Vibrio cholerae*
ab2015-06-02-20	2524250ba11140c386c211aa14d164a166926<br>ee9ca7af5566eedbbba29856c48	*Vibrio cholerae*
ab2015-06-02-21	95a8202f59bdcfc9814f417aa40b3c355fc6f12c<br>b147784d1d1665d8ccce8221	*Vibrio cholerae*
ab2015-06-02-22	09443b1917375d0f2755c3f4c2029dc847c654<br>efdb2e5a3df0c5938a9afaa1a9	*Vibrio cholerae*

Jensen Lab id	OIP id	Organism
ab2015-06-02-23	453536f21d3074fd3f6fcae6d834c6e37335f46 6611e2f1842c6beb8ee77681e	*Vibrio cholerae*
ab2015-06-02-24	03ab91b142bf62ed9c21e1da4b17e25d7c73b7 0cbd6a25481342d9736f2a2290	*Vibrio cholerae*
ab2015-06-02-25	8853aea7df6d8a168b62b9db8694ad82dfa23c 9b00eafb3f71b025f20cb34abd	*Vibrio cholerae*
ab2015-06-02-26	8770b212ceddc32db6f1ae0b8a40a5eaa1ec42c 31bceda0c4919b86dcde7246b	*Vibrio cholerae*
ab2015-06-02-27	e1b34b3df7aa6367398010bf8b96d5a003759a 2dd71a473e45a43d598e779d29	*Vibrio cholerae*
ab2015-06-02-29	08324bf01302b1db927045548218ff3e317aea e74602363e669a187c52e234be	*Vibrio cholerae*
ab2015-06-02-30	2eda58ca01240b7486bda36c36e36217918c8 9fcb1361ddda13eeda95afc4271	*Vibrio cholerae*
ab2015-06-02-31	6b6b8ba7f2127c66614756b85d4bb0efde943c bfc305e40a3330181009bd893e	*Vibrio cholerae*
ab2015-06-02-32	61c86c9a912eb1bcada440939a48d46e99128f feca99578b16e00e22031f4a22	*Vibrio cholerae*
ab2015-06-03-1	197f9f4bbc9f5fa49c4644f3f40df46cbf2b82399 e8137db7799b9e030bc8455	*Vibrio cholerae*
ab2015-06-03-2	544970e8e49ff409d4f61730c89757fdbee6fff3 64c1c604d355bc522c8d0f12	*Vibrio cholerae*
ab2015-06-03-3	e87e0b6e154e5dfec0120ce92e6400764ae249 64aa592553e49de6187b7ffb57	*Vibrio cholerae*
ab2015-06-03-4	20d581e5578e3156ddf96496119c1296494e0 62deecf45634c79f19f331368b6	*Vibrio cholerae*
ab2015-06-03-5	10c8a0be7b87b3d95eb2d8573e782a856e493 b65735aab290c0a772092d2ecfc	*Vibrio cholerae*
ab2015-06-03-6	0c1b01aa1ed45fcba7d17500c4829f9480924fa 210f9706d6a1a5da9d4dc1477	*Vibrio cholerae*
ab2015-06-03-7	21482339f240527367c699214ba438d2b9a7a c6376d611f2d69e0f4c1d1d0039	*Vibrio cholerae*
ab2015-06-03-8	453a055bebf6eb8d65f2154f51a568cfc3f58c36 aa445ff6be7a820c0d3963e9	*Vibrio cholerae*
ab2015-06-03-9	faed3ad567cb4667d1986bb9a7869a4a9da0a9 e2d016dbb6d823e15f5d1cc149	*Vibrio cholerae*

Jensen Lab id	OIP id	Organism
ab2015-06-03-10	1c8abd7e25085cccce2ce38fb25ff51ac70ddfc88cad18322406467298bfaa3e	*Vibrio cholerae*
ab2015-06-03-11	920321536f97c55c300ad77d279265973442bd4ec0e7286a95f2d134ec384b42	*Vibrio cholerae*
ab2015-06-03-12	9262dbfb593f79bf0c9cac6f9125326ede755dd3e4edc569b68b0fc279f84d8d	*Vibrio cholerae*
ab2015-06-03-13	72b627c7010d0ea37a8aa011925069437195c51f77e5adcf41f6a8330e75cc65	*Vibrio cholerae*
ab2015-06-03-14	223d692a658a507a7085c75f8c757b05c1cf1d2ee8e352a1db4371f2062e3ec4	*Vibrio cholerae*
ab2015-06-03-15	c55cf52f955779f23cda1c2244e2b64315f8fbb490f10689f9c32b7a87a99512	*Vibrio cholerae*
ab2015-06-03-16	b59e667fadb1914c70ad191dad31a30a87ba416e8de4877000d1f6cb52869601	*Vibrio cholerae*
ab2015-06-03-17	067ac5b8ce49dffe5254c958a0ef6bfd601de8778c47f17d8db6c76f2b03d882	*Vibrio cholerae*
ab2015-06-03-18	c9dd866db34f7ed34af143d2a879e5b28035fce2a4392aceb54f7824ceec0e37	*Vibrio cholerae*
ab2015-06-03-19	fabd724070997b695344be1bc4ad91faa2b2c82876f6bda65aeeee18eb39ca29	*Vibrio cholerae*
ab2015-06-03-20	34ff16d0af7b7a6cfbe49d20981049070d2b9b9f5a1919896a839eb62dc8c6a8	*Vibrio cholerae*
ab2015-06-03-21	4440060c9b511b30ec0b7c123b3891a3823a77a8393437862aa5344392e0be26	*Vibrio cholerae*
ab2015-06-03-22	48c976b5ec236342fdd4aefe521086e3657da4a45c4afad6003efdb44a5e8275	*Vibrio cholerae*
ab2015-06-03-23	1c1e69acb1c51ec5655f2fc7e1296de6494c1c68785e7f4a7ce0cc651d2125c9	*Vibrio cholerae*
ab2015-06-03-24	aa070936e1aee30b373bc2d2aed88f1576cd3ed6766dbc9f92f120be46aeeabe	*Vibrio cholerae*
ab2015-06-03-25	d84b38caf03c63be65666a628fa8f90aeaec8991ba29dad0b539585fbcf3b96c	*Vibrio cholerae*
ab2015-06-03-26	f38ac8c272b532e7dd3e3da38b1fff38f42385a2aa55872dda24bf10720544ba	*Vibrio cholerae*
ab2015-06-03-27	9d8070fbfbab0e5bfdd555544219789104fb5dcbda613d24385c416ccd3c3158	*Vibrio cholerae*

Jensen Lab id	OIP id	Organism
ab2015-06-03-28	bd0aaf3c9bb4904c623c2304b5ca71f3e7dbbb d15fdbe2eb481dbdb01d561986	*Vibrio cholerae*
ab2015-06-03-29	81345effbcb7600a9f57a2f556a10f3a20a6845 df3ca51ccc1d67d964ee8e21f	*Vibrio cholerae*
ab2015-06-04-5	e95c286a1ca5b5b3b3b5bf0a318a30c627623d 2b63b68c9991dab337ae48e8c2	*Vibrio cholerae*
ab2015-06-04-6	79b0258243f18a1752dac35aa7e0bff1a711f79 1ddd0379c184e05d9d1c5dd86	*Vibrio cholerae*
ab2015-06-04-7	6b8f4ae3d17ab82176fe1bb2c79c55d5ce757c 5355323bbadc84d17a5a6f75dd	*Vibrio cholerae*
ab2015-06-04-8	18fca4c49057b64129c9dbd0f9c8be7562fcee4 d5fff21a449b1fd79a5e910f8	*Vibrio cholerae*
ab2015-06-04-9	a9e50ed3ce23097a99fdaa4d949f5353bcba9f5 ce3cd7a49d5f3df08d668a989	*Vibrio cholerae*
ab2015-06-04-10	275fbd79f1fb013309e3dc4a22c607ab406b6b 2a16489a508c3490ede71dac93	*Vibrio cholerae*
ab2015-06-04-11	eadc30e57a5bbceba8e69544d43debdb4d7139 2ef20f16c8d9a5659a2dc877a9	*Vibrio cholerae*
ab2015-06-04-12	82b9550d3ebda9a078ece5cea3cfd02ceece06d f9115d8b2d558d5b5c073f9dd	*Vibrio cholerae*
ab2015-06-04-13	204dea6006208b691ae45b1aa8a62421894c0 d956a5d5bf6aaa8a3e75253345d	*Vibrio cholerae*
ab2015-06-04-14	7504bd89ddb0fd83ed926065c9d2cf7b57db00 72ebb740cfefdc957f1d5c4c81	*Vibrio cholerae*
ab2015-06-04-15	7bc4afbf390105748d85295198579497fd0d67 12da0eb190eec2436cf6617349	*Vibrio cholerae*
ab2015-06-04-16	c99f6e5934554670fe2166f2f8deb7ad17f4536 96c914b4ca1ea88f7e009f0a4	*Vibrio cholerae*
ab2015-06-04-17	095901997ad173f1a9cf5804cd7f92e2c95ee7b d4be14b1fa1fe94ed74e6a85d	*Vibrio cholerae*
ab2015-06-04-18	2f236dac0a263b2e3278aef8ab2aad6c7ace83b de857c6916683146369424e93	*Vibrio cholerae*
ab2015-06-04-19	b4daca6e8f77b714d01faac2b7dc9933c4617d 736b0a6ef29eb9e1056c0214f8	*Vibrio cholerae*
ab2015-06-04-20	ed95dd31b7e3a97a2bedc0e8985ad471dad7c7 447a7070d5d5b37b8f259da41a	*Vibrio cholerae*

Jensen Lab id	OIP id	Organism
ab2015-06-04-21	a41257cc5f1d3c4a84cd9645921671bff4e5b58 de593872740a35172fd4102a5	*Vibrio cholerae*
ab2015-06-04-22	863a029fcd72521b29fa741b7a13b2df4420fcc 9491a090eab709c7c7d727514	*Vibrio cholerae*
ab2015-06-04-23	c63edc39ab954f54d3ec80607005b54739f694 0f8e317c3ea88d951b0e63ed82	*Vibrio cholerae*
ab2015-06-04-24	5f040233e108f1d0d81448d4b7c44476947e2 075742fd79ad2f0d0adf9f686fa	*Vibrio cholerae*
ab2015-06-04-25	9a31271d398507a3e7cb0694927dbda47043f 35bf8171cbd4f56df5896c7e060	*Vibrio cholerae*
ab2015-06-04-26	687d83b774894a40b9f35c54e0e50d2e11613 d6260723ab83573e3fd6c55b309	*Vibrio cholerae*
ab2015-06-04-27	5cc452dfbe2e22d09dd2102b010e926f7c9735 5c0c209148f049292cf3b910de	*Vibrio cholerae*
ab2015-06-04-28	49ffb9f6a24a7d07799479b844a01ed91a204f9 5afa28a8d59561b858d9a4d19	*Vibrio cholerae*
ab2015-06-04-29	38f8ed8a6cba9fdab947246fd283023b21353f1 66c6e41f949320a028cea2f1a	*Vibrio cholerae*
ab2015-06-04-30	92c05f4866a31ee526bac1d95d513c9d0063e7 94554cdc124651fabc1e8c9395	*Vibrio cholerae*
ab2015-06-04-31	60d87f38e11916f13cf6345b22f0b5fc567d6ec ba651e7ed98c1ba23faca1a27	*Vibrio cholerae*
ab2015-06-04-32	2b200698dc605f2234c28d33d12a2eeed4bfb6 808399563791bf228fda34936e	*Vibrio cholerae*
ab2015-05-29-38	71b7016135114ee9988faf24a00aec627349d8 610625a8925152e418ce042cb8	*Pseudomonas aeruginosa*
ab2015-05-29-33	3bc9419d595c59d2ffb177294ceb038f5c4a402 87cc1d2ff95ab43e42e22c5b5	*Pseudomonas aeruginosa*
ab2015-05-29-34	baf59323b4cd6b20e471b0587f4349bb437cc8 9feba37eb726c76d2b35996859	*Pseudomonas aeruginosa*
ab2015-05-29-35	375ec0e6c3542bc3d33e7ec94c751bd1669ffe9 984a4ccb8948ddc246bd02517	*Pseudomonas aeruginosa*
ab2015-05-29-36	dda73882e77f51644ba4d9fc73810f58c0789cf 5ebd5c4c64108940c978d3913	*Pseudomonas aeruginosa*
ab2015-05-29-37	e0d1b4daa89125bfae9539157ab0d459e9a10b 53148d17af60eb3afadf667e37	*Pseudomonas aeruginosa*

Jensen Lab id	OIP id	Organism
ab2015-05-29-38	71b7016135114ee9988faf24a00aec627349d8 610625a8925152e418ce042cb8	*Pseudomonas aeruginosa*
ab2015-05-29-39	9ff1533aa7d28d3d64439293ea263ca3ce6a61 01f19c7367426d9a3bba3773e6	*Pseudomonas aeruginosa*
ab2015-05-29-40	c5f0fcf46599f6ee48fe23a59fc1d752cd64ab40 31c3846ffb85d078a8bfaf5c	*Pseudomonas aeruginosa*
ab2015-05-29-41	fe52314687302e52d76a76292a84a8e7730dac 37451c7026621190f2185cd7df	*Pseudomonas aeruginosa*
ab2015-05-29-42	fc8e9658e5fe5d5e30364f389fc123f474537bd a5132fb71b12af99aaf02e7a2	*Pseudomonas aeruginosa*
ab2015-05-29-43	433cf08b0ab2269a91f4e946b17c551e8a92a3 0c8c5214bc8384072fc8915b85	*Pseudomonas aeruginosa*
ab2015-05-29-44	9162280a2e505e7a74c03de6202f084eb4b838 82453525539fdf87da3905d77c	*Pseudomonas aeruginosa*
ab2015-05-29-45	9db0388f6a5482eb6cad84cd7abf0ddad7bcd4 a3d741d971cc9dd3a2c378cda2	*Pseudomonas aeruginosa*
ab2015-05-29-46	31eee1b22c3f3010a3a31df973be75ff1a12606 9e78bfbb1dbed30cd3985330d	*Pseudomonas aeruginosa*
ab2015-05-29-47	799edd343b37e6dff51118d39052ad7241e7c0 5238286bcc779c1ab9227f8a4c	*Pseudomonas aeruginosa*
ab2015-05-29-48	72025551790f3ada513cffe56e1f0971b3844f1 c985e4f3e3b7579dd349aecd1	*Pseudomonas aeruginosa*
ab2015-05-29-49	76b1c8f739812662c22aee910f2e1a2bc7ac72f 9c79ee19df0493c60be7f6711	*Pseudomonas aeruginosa*
ab2015-05-29-50	2f3db7b47e7d42594be348214e07578d7ea1f0 8a10f8cd937bec3abb8692c6aa	*Pseudomonas aeruginosa*
ab2015-05-29-51	dbe07cf9023a7c8d67cea0f6bc02eeaea93f5c2e 60453ca2aa4685d8e3a53654	*Pseudomonas aeruginosa*
ab2015-05-29-52	8f861e2f49e3b2155f99422428acb7ca764c5c2 2352dd6f5c411d89daaa99c8c	*Pseudomonas aeruginosa*
ab2015-05-29-53	8bbc0699df1ecaeff06813ea816ded484d90867 9412e938d42afbb30e0f47747	*Pseudomonas aeruginosa*
ab2015-05-29-54	87be5d50a167d3c23ad2126bc4d72844c0ed6 af2848768fd1bb764ad0a05355b	*Pseudomonas aeruginosa*
ab2015-05-29-55	975a50a6ac4a0a1ce191d3f339c433b73ba15a 3a8eff22b3768a9327e1ca5a21	*Pseudomonas aeruginosa*

Jensen Lab id	OIP id	Organism
ab2015-05-29-56	d0799bdb371912d4370d6226ae8263e1f9bf4 19c49c42c16514c657f11f05f37	*Pseudomonas aeruginosa*
ab2015-05-29-57	dfd11ff2b7b5261968452f994235bece1d208b 793f0c7768b883163fa0cad72c	*Pseudomonas aeruginosa*
ab2015-05-29-58	06dc2aa14271a7d5c79c6e112a3aeacc8c6432 46c659fab33b3bbb01fbe25356	*Pseudomonas aeruginosa*
ab2015-05-29-59	f92bcceb78dac999467e57653d9e3ab4017261 8fc09fb5806e64040e8a3410f5	*Pseudomonas aeruginosa*
ab2015-05-29-60	bd0b6b93ba4f3528fe3f78a789dd847ee83a6d 4b08cd6e652053e72aefa6897b	*Pseudomonas aeruginosa*
ab2015-05-29-61	37d4d1c6d5229a5db3700596859b305aea3aa 460d67362ba176424c9217ebf28	*Pseudomonas aeruginosa*
ab2015-05-29-62	4e059367636fa821409e894b49151556c01d6 5ebe01eca3e4d4fedec6531adc7	*Pseudomonas aeruginosa*
ab2014-010-14-1	b9d6746d36ee7943e0eb19c9299214e60080c 78b367558d6748820c84c2aec76	*Pseudomonas aeruginosa*
ab2014-010-14-10	c8c1989c09901dd7a257d86497a713e85a761 edd2209efe0d8784d228e5b3585	*Pseudomonas aeruginosa*
ab2014-010-14-11	18ec447aac947d6ed610084fbb9e808bdb761b f68919b853ac4946632d6c6e50	*Pseudomonas aeruginosa*
ab2014-010-14-12	58c0a2aab1b8f0322fcc4b93838458a820fc8db 74f716b53ba166ae2cbe92001	*Pseudomonas aeruginosa*
ab2014-010-14-13	9e8108ef3cf66406b628ec973b10079efc0ca57 81a05ca1a875ed6fdecc2fe02	*Pseudomonas aeruginosa*
ab2014-010-14-14	7935084f249ec5bc222b38951cd369dab76cfa b95a769d42d0eb1312cc5bb50c	*Pseudomonas aeruginosa*
ab2014-010-14-15	51b27c3cf74ce394c1d54773bd1a7d15d3379f c8db4aa2f8b9dbdbaabdd71251	*Pseudomonas aeruginosa*
ab2014-010-14-17	764f7220c7323438d65b7d4334916e9aadb0b e04be1b662615be1ad1312407c6	*Pseudomonas aeruginosa*
ab2014-010-14-22	797a818b0722e811f5ed0d6d22bdfdb158fabb cd22e8bfc7f1010ad265af8897	*Pseudomonas aeruginosa*
ab2014-010-14-23	322a5ff8d6dda5854aaafec4883226c8463c2e9 a08ce553c969ca6b98cfedef9	*Pseudomonas aeruginosa*
ab2014-010-14-3	2c11ded42bbc0cd87dc67464474187c3c82d7 1742a8b50f346516a958361706a	*Pseudomonas aeruginosa*

Jensen Lab id	OIP id	Organism
ab2014-010-14-4	20cd19c0fa4b3e32b33075348be5ab3102def5 083cce6fd7ac55b01ac0cb5835	*Pseudomonas aeruginosa*
ab2014-010-14-5	3e6133278fc31ba361c1cd7c95c081a822d972 3dc3b7f3549870510a422e2522	*Pseudomonas aeruginosa*
ab2014-010-14-6	3cbeac672c18cd66252b47d65f0ec124b17baf5 19849a6844fab4af70fbbcea1	*Pseudomonas aeruginosa*
ab2014-010-14-8	cb237b8021a9e653613163394c11258bb3b72 fe09720a977596c18478e5d43c2	*Pseudomonas aeruginosa*
ab2014-010-14-9	8bf9ec4e43a3e6bd11c44bb6d341d138e14b41 c776e66e21a8a17d399c1c69c2	*Pseudomonas aeruginosa*
sc2014-02-14-1	ecfe996b6c698beb104f0cf84bda7955a48c7ed b6049ce2b9256cb3b1e2b85b0	*Methylomicrobium alcaliphylum*
sc2014-02-12-1	37a38f6896b2fbc3379ebba9976e69ccefd45da a011b009f9fc498f40ab5b68e	*Methylomicrobium alcaliphylum*
sc2014-02-12-3	82c7e7111fad00bd9de9be253c966a0d87b3b5 85cf47a4e3ada52b7a90ae362f	*Methylomicrobium alcaliphylum*
sc2014-02-12-6	c807ab328c5bd06874ad4f9fb731fafd888f238 94d00cafed1089f988407ed99	*Methylomicrobium alcaliphylum*
sc2014-02-12-7	99a62dfe3b6bedf92f6bd93916b1fe055c1abd7 d4d2f781348c01ae89c45dbd5	*Methylomicrobium alcaliphylum*
sc2014-02-12-8	6ce0a323a040374567356e08c438fd42806aef 88c73f105e2d9e13684d940ae8	*Methylomicrobium alcaliphylum*
sc2014-02-12-9	9e828a83fb3538f1f6f9942ea4216cd7fe20ebcc 1c82ed3dba8f7e34bdc3a1e9	*Methylomicrobium alcaliphylum*
sc2014-02-12-12	262b60a13fbcd486cc7c4ac73b739ddccb23d9 3cf8d5d4138ae911af40f95480	*Methylomicrobium alcaliphylum*

Supplementary Table 7: Atomic models used to produce the homology models used in this work

Domain(s)	PDB code	Reference
HAMP(*A. fulgidus*) + MCP_Signal(*E. coli*)	3ZX6	[21]
PAS(*P. aeruginosa*)	4HI4	[22]
PAS + HAMP (*P. aeruginosa*)	3VOL	[23]

Domain(s)	PDB code	Reference
3xHAMP (*P. aeruginosa*)	4I3M	[24]

Supplementary Table 8: Relevant files used to build the homology models produced in this work

File	Type	Description
3XZ6_4I3M.pir	Sequence alignment	Sequence alignment used to build the 2H+S homology model
3XZ6_4I3M_74.pdb	3D atomic model	Best homology model from 2H+S
4HI4_BD.pdb	3D atomic model	chains B and D of 4HI4 aligned with 2H+S model
3ZX6_4I3M_4HIH.pir	Sequence alignment	Sequence alignment used to build the P+2H+S homology model
3ZX6_4I3M_4HIH_99.pdb	3D atomic model	Best homology model from P+2H+S
4I3M.bio.pos.pdb	3D atomic model	Model of 4I3M positioned against 3ZX6_4I3M_4HIH_99.pdb to build the model for Aer2 (PA0176)
3ZX6_4I3M_4HI4_4I3M.pir	Sequence alignment	Sequence alignment used to build the model for Aer2 (PA0176)
Aer2Pa_3HAMP_PAS_2HAMP.B99990041.pdb	3D atomic model	Best Aer2 (PA0176) homology model
3ZX6_4I3M_4HI4_4HI4.pir	Sequence alignment	Sequence alignment used to build the model for Aer2-like (VCA1092)
VCA1092.B999900035.pdb	3D atomic model	Best Aer2-like (VCA1092) homology model
3ZX6_4I3M_4HI4_4HI4_SO.pir	Sequence alignment	

File	Type	Description
	used to build the model for Aer2-like (SO_2123)	
SO_2123.B99990017.pdb	3D atomic model	Best Aer2-like (SO_2123) homology model
hamp_sequence_for_MEALZ.linsi.fa	Sequence alignment	Sequence alignment of HAMP domains in the group of Pseudomonas group similar to the 3 HAMPs in 4I3M and the C-terminal HAMP of MEALZ_2872
RAxML_bipartitions_50coll.hamp_sequence_for_MEALZ.linsi.rec.tree	Phylogenetic Tree	The tree with maximum likelihood based on hamp_sequence_for_MEALZ.linsi.fa
4I3M.bio.HAMP2.withtail4alignment.pdb	3D atomic model	Model of the second HAMP of 4I3M with part of the helix connecting to the third HAMP.
4I3M.bio.HAMP2.alnMEALZ.pdb	2\3D atomic model	Model of the second HAMP of 4I3M without part of the helix connecting to the third HAMP.
3ZX6_4I3M_4HI4_HAMP2_MEALZ.pir	Sequence alignment used to build the model for Aer2-like (MEALZ_2872)	
MEALZ_2872_wHAMP.B99990020.pdb	3D atomic model	Best Aer2-like (MEALZ_2872) homology model

Supplementary Table 9: 310 randomly selected non-redundant γ-Proteobacteria genomes used in this work. The presence of an F7 system is indicated.

**Genome**	**has F7**
Acidithiobacillus caldus SM-1	no
Acidithiobacillus ferrivorans SS3	no
Acidithiobacillus sp. GGI-221	no
Acidithiobacillus thiooxidans ATCC 19377	no
Acinetobacter baumannii AB5075	no
Acinetobacter bereziniae LMG 1003	no
Acinetobacter calcoaceticus RUH2202	no
Acinetobacter haemolyticus ATCC 19194	no
Acinetobacter johnsonii SH046	no
Acinetobacter junii SH205	no
Acinetobacter lwoffii SH145	no
Acinetobacter nosocomialis Ab22222	no
Acinetobacter oleivorans DR1	no
Acinetobacter parvus DSM 16617 = CIP 108168	no
Acinetobacter radioresistens DSM 6976 = NBRC 102413	no
Acinetobacter sp. NCTC 10304	no
Acinetobacter ursingii DSM 16037 = CIP 107286	no
Aeromonas aquariorum AAK1	no
Aeromonas caviae Ae398	no
Aeromonas hydrophila SSU	no
Aeromonas media WS	no
Aeromonas salmonicida subsp. salmonicida A449	no
Aeromonas veronii AER397	no
Alcanivorax borkumensis SK2	no
Alcanivorax dieselolei B5	yes
Alcanivorax hongdengensis A-11-3	no

**Genome**	**has F7**
Alcanivorax pacificus W11-5	yes
Alcanivorax sp. DG881	no
Aliivibrio salmonicida LFI1238	no
Alishewanella aestuarii B11	no
Alishewanella agri BL06	no
Alishewanella jeotgali KCTC 22429	no
Alkalilimnicola ehrlichii MLHE-1	no
Allochromatium vinosum DSM 180	yes
Alteromonadales bacterium TW-7	yes
Alteromonas mediterranea MED64	no
Alteromonas sp. SN2	yes
Azotobacter vinelandii DJ	yes
Beggiatoa alba B18LD	yes
Beggiatoa sp. SS	yes
Cardiobacterium hominis ATCC 15826	no
Cardiobacterium valvarum F0432	no
Cellvibrio japonicus Ueda107	no
Cellvibrio sp. BR	yes
Chromohalobacter salexigens DSM 3043	yes
Citrobacter freundii 4_7_47CFAA	yes
Citrobacter koseri ATCC BAA-895	yes
Citrobacter rodentium ICC168	yes
Citrobacter sp. 30_2	yes
Citrobacter youngae ATCC 29220	yes
Colwellia psychrerythraea 34H	no
Cronobacter sakazakii ES15	yes

**Genome**	**has F7**
Cronobacter turicensis z3032	yes
Dichelobacter nodosus VCS1703A	no
Dickeya dadantii Ech703	yes
Dickeya zeae Ech1591	yes
Ectothiorhodospira sp. PHS-1	yes
Edwardsiella ictaluri 93-146	yes
Edwardsiella tarda ATCC 23685	yes
Endoriftia persephone 'Hot96_1+Hot96_2'	no
Enhydrobacter aerosaccus SK60	no
Enterobacter asburiae LF7a	yes
Enterobacter cancerogenus ATCC 35316	yes
Enterobacter cloacae subsp. cloacae GS1	yes
Enterobacter hormaechei ATCC 49162	yes
Enterobacter radicincitans DSM 16656	yes
Enterobacter sp. 638	yes
Enterobacteriaceae bacterium 9_2_54FAA	yes
Erwinia amylovora CFBP1430	yes
Erwinia billingiae Eb661	yes
Erwinia pyrifoliae Ep1/96	yes
Erwinia sp. Ejp617	yes
Erwinia tasmaniensis Et1/99	yes
Escherichia albertii TW11588	yes
Escherichia coli KTE229	yes
Escherichia fergusonii ECD227	yes
Escherichia hermannii NBRC 105704	yes
Escherichia sp. TW09276	yes

Genome	has F7
Ferrimonas balearica DSM 9799	no
Fluoribacter dumoffii Tex-KL	no
Frateuria aurantia DSM 6220	yes
Gallaecimonas xiamenensis 3-C-1	yes
Glaciecola agarilytica NO2	yes
Glaciecola arctica BSs20135	no
Glaciecola chathamensis S18K6	yes
Glaciecola lipolytica E3	no
Glaciecola mesophila KMM 241	no
Glaciecola nitratireducens FR1064	yes
Glaciecola pallidula DSM 14239 = ACAM 615	yes
Glaciecola polaris LMG 21857	no
Glaciecola psychrophila 170	no
Glaciecola sp. 4H-3-7+YE-5	yes
Grimontia hollisae CIP 101886	yes
Grimontia sp. AK16	yes
Hafnia alvei ATCC 51873	yes
Hahella chejuensis KCTC 2396	yes
Halomonas boliviensis LC1	yes
Halomonas elongata DSM 2581	yes
Halomonas sp. GFAJ-1	yes
Halomonas titanicae BH1	yes
Halorhodospira halophila SL1	yes
Halothiobacillus neapolitanus c2	no
Hydrocarboniphaga effusa AP103	yes
Idiomarina loihiensis L2TR	no

**Genome**	**has F7**
Idiomarina xiamenensis 10-D-4	no
Kangiella koreensis DSM 16069	no
Klebsiella aerogenes KCTC 2190	yes
Klebsiella pneumoniae subsp. pneumoniae HS11286	no
Legionella drancourtii LLAP12	yes
Legionella longbeachae NSW150	no
Legionella pneumophila subsp. pneumophila	no
Listonella anguillarum M3	yes
Marichromatium purpuratum 984	yes
Marinobacter adhaerens HP15	no
Marinobacter algicola DG893	no
Marinobacter hydrocarbonoclasticus ATCC 49840	no
Marinobacter hydrocarbonoclasticus VT8	no
Marinobacter manganoxydans MnI7-9	no
Marinobacter santoriniensis NKSG1	no
Marinobacter sp. ELB17	no
Marinobacterium stanieri S30	yes
Marinomonas mediterranea MMB-1	yes
Marinomonas posidonica IVIA-Po-181	no
Marinomonas sp. MWYL1	yes
Methylobacter tundripaludum SV96	yes
Methylococcus capsulatus str. Bath	no
Methylomicrobium album BG8	yes
Methylomicrobium alcaliphilum 20Z	yes
Methylomonas methanica MC09	yes
Methylophaga aminisulfidivorans MP	yes

Genome	has F7
Methylophaga frappieri	no
Methylophaga lonarensis MPL	no
Methylophaga thiooxydans DMS010	no
Moraxella macacae 0408225	no
Morganella morganii subsp. morganii KT	yes
Moritella sp. PE36	yes
Nitrosococcus halophilus Nc 4	no
Nitrosococcus oceani ATCC 19707	no
Nitrosococcus watsonii C-113	no
Oceanimonas sp. GK1	no
Pantoea agglomerans 299R	yes
Pantoea ananatis LMG 20103	yes
Pantoea sp. aB	yes
Pantoea stewartii subsp. stewartii DC283	yes
Pantoea vagans C9-1	yes
Pectobacterium atrosepticum SCRI1043	yes
Pectobacterium carotovorum subsp. brasiliensis PBR1692	yes
Pectobacterium sp. SCC3193	yes
Pectobacterium wasabiae CFBP 3304	yes
Photobacterium damselae subsp. damselae CIP 102761	no
Photobacterium leiognathi subsp. mandapamensis svers.1.1.	no
Photobacterium profundum SS9	no
Photobacterium sp. AK15	no
Photorhabdus asymbiotica	yes
Photorhabdus luminescens subsp. laumondii TTO1	yes
Proteus mirabilis WGLW6	yes

Genome	has F7
Proteus penneri ATCC 35198	yes
Providencia alcalifaciens DSM 30120	yes
Providencia burhodogranariea DSM 19968	yes
Providencia rettgeri Dmel1	no
Providencia rustigianii DSM 4541	yes
Providencia stuartii ATCC 25827	yes
Pseudoalteromonas arctica A 37-1-2	no
Pseudoalteromonas atlantica T6c	no
Pseudoalteromonas citrea NCIMB 1889	yes
Pseudoalteromonas haloplanktis ANT/505	yes
Pseudoalteromonas luteoviolacea B = ATCC 29581	yes
Pseudoalteromonas marina mano4	yes
Pseudoalteromonas piscicida JCM 20779	yes
Pseudoalteromonas rubra ATCC 29570	yes
Pseudoalteromonas sp. Bsw20308	yes
Pseudoalteromonas spongiae UST010723-006	yes
Pseudoalteromonas undina NCIMB 2128	yes
Pseudomonas aeruginosa LESB58	yes
Pseudomonas avellanae BPIC 631	no
Pseudomonas brassicacearum subsp. brassicacearum NFM421	no
Pseudomonas denitrificans ATCC 13867	yes
Pseudomonas entomophila L48	no
Pseudomonas extremaustralis 14-3 substr. 14-3b	no
Pseudomonas fluorescens F113	no
Pseudomonas fragi A22	no
Pseudomonas fulva 12-X	no

**Genome**	**has F7**
Pseudomonas fuscovaginae UPB0736	no
Pseudomonas geniculata N1	yes
Pseudomonas mendocina ymp	no
Pseudomonas monteilii SB3078	no
*Pseudomonas poae RE*1-1-14*	no
Pseudomonas protegens CHA0	no
Pseudomonas pseudoalcaligenes KF707	yes
Pseudomonas psychrotolerans L19	no
Pseudomonas putida GB-1	no
Pseudomonas resinovorans NBRC 106553	yes
Pseudomonas sp. TKP	no
Pseudomonas stutzeri KOS6	no
Pseudomonas syringae pv. phaseolicola 1448A	no
Pseudomonas viridiflava UASWS0038	no
Pseudoxanthomonas spadix BD-a59	no
Pseudoxanthomonas suwonensis 11-1	yes
Psychrobacter arcticus 273-4	no
Psychrobacter cryohalolentis K5	no
Psychrobacter sp. PRwf-1	no
Psychromonas sp. CNPT3	no
Rahnella aquatilis CIP 78.65 = ATCC 33071	yes
Rahnella sp. Y9602	yes
Rheinheimera nanhaiensis E407-8	no
Rheinheimera sp. A13L	no
Rhodanobacter fulvus Jip2	yes
Rhodanobacter sp. 116-2	no

Genome	has F7
Rhodanobacter spathiphylli B39	no
Rhodanobacter thiooxydans LCS2	yes
Saccharophagus degradans 2-40	yes
Salinisphaera shabanensis E1L3A	yes
Salmonella bongori N268-08	yes
Salmonella enterica subsp. enterica serovar Gallinarum str. 9184	yes
Serratia liquefaciens ATCC 27592	yes
Serratia marcescens VGH107	yes
Serratia odorifera 4Rx13	yes
Serratia plymuthica S13	yes
Serratia proteamaculans 568	yes
Serratia sp. AS13	yes
Shewanella amazonensis SB2B	yes
Shewanella baltica OS155	yes
Shewanella benthica KT99	yes
Shewanella denitrificans OS217	no
Shewanella frigidimarina NCIMB 400	no
Shewanella halifaxensis HAW-EB4	no
Shewanella loihica PV-4	yes
Shewanella oneidensis MR-1	yes
Shewanella pealeana ATCC 700345	no
Shewanella piezotolerans WP3	no
Shewanella putrefaciens CN-32	no
Shewanella sediminis HAW-EB3	yes
Shewanella sp. MR-4	yes
Shewanella violacea DSS12	yes

Genome	has F7
Shewanella woodyi ATCC 51908	yes
Shigella boydii CDC 3083-94	yes
Shigella dysenteriae 1617	no
Shigella flexneri 4343-70	yes
Shigella sonnei Ss046	yes
Shigella sp. D9	yes
Simiduia agarivorans SA1 = DSM 21679	yes
Stenotrophomonas maltophilia K279a	yes
Stenotrophomonas sp. SKA14	yes
Teredinibacter turnerae T7901	yes
Thalassolituus oleivorans MIL-1	yes
Thioalkalimicrobium aerophilum AL3	yes
Thioalkalivibrio sp. K90mix	no
Thioalkalivibrio sulfidiphilus HL-EbGr7	no
Thiocapsa marina 5811	no
Thiocystis violascens DSM 198	yes
Thiomicrospira crunogena XCL-2	yes
Thiorhodococcus drewsii AZ1	yes
Thiorhodospira sibirica ATCC 700588	yes
Thiorhodovibrio sp. 970	no
Thiothrix nivea DSM 5205	no
Vibrio alginolyticus 40B	no
Vibrio anguillarum 775	yes
Vibrio brasiliensis LMG 20546	yes
Vibrio campbellii CAIM 519 = NBRC 15631	no
Vibrio caribbenthicus ATCC BAA-2122	no

Genome	has F7
Vibrio cholerae HC-23A1	no
Vibrio coralliilyticus ATCC BAA-450	yes
Vibrio fischeri MJ11	no
Vibrio furnissii CIP 102972	yes
Vibrio harveyi 1DA3	no
Vibrio ichthyoenteri ATCC 700023	no
Vibrio metschnikovii CIP 69.14	no
Vibrio mimicus MB451	yes
Vibrio nigripulchritudo ATCC 27043	yes
Vibrio ordalii ATCC 33509	yes
Vibrio orientalis CIP 102891 = ATCC 33934	yes
Vibrio parahaemolyticus O1:Kuk str. FDA_R31	no
Vibrio rotiferianus DAT722	no
Vibrio scophthalmi LMG 19158	no
Vibrio shilonii AK1	no
Vibrio sinaloensis DSM 21326	yes
Vibrio sp. HENC-01	no
Vibrio splendidus ATCC 33789	no
Vibrio tubiashii NCIMB 1337 = ATCC 19106	yes
Vibrio vulnificus MO6-24/O	yes
Vibrionales bacterium SWAT-3	no
Wohlfahrtiimonas chitiniclastica SH04	no
Xanthomonas albilineans GPE PC73	yes
Xanthomonas axonopodis pv. malvacearum str. GSPB2388	yes
Xanthomonas campestris pv. musacearum NCPPB 4381	yes
Xanthomonas citri subsp. citri Aw12879	yes

Genome	has F7
Xanthomonas fuscans subsp. aurantifolii str. ICPB 10535	yes
Xanthomonas gardneri ATCC 19865	yes
Xanthomonas oryzae pv. oryzicola BLS256	yes
Xanthomonas perforans 91-118	yes
Xanthomonas sacchari NCPPB 4393	yes
Xanthomonas translucens DAR61454	yes
Xanthomonas vesicatoria ATCC 35937	yes
Xenorhabdus bovienii SS-2004	yes
Xenorhabdus nematophila ATCC 19061	yes
Xylella fastidiosa Temecula1	no
Yersinia aldovae ATCC 35236	yes
Yersinia bercovieri ATCC 43970	yes
Yersinia enterocolitica subsp. palearctica Y11	yes
Yersinia frederiksenii ATCC 33641	yes
Yersinia intermedia ATCC 29909	yes
Yersinia kristensenii ATCC 33638	yes
Yersinia mollaretii ATCC 43969	yes
Yersinia pestis PY-16	yes
Yersinia pseudotuberculosis PB1/+	yes
Yersinia rohdei ATCC 43380	yes
Yersinia ruckeri ATCC 29473	yes
Yokenella regensburgei ATCC 43003	yes
endosymbiont of Riftia pachyptila (vent Ph05)	yes
gamma proteobacterium HdN1	no

Supplementary Table 10: Genomes used in phylogenetic profiles.

**Genomes imaged in this study**
Methylomicrobium alcaliphilum 20Z
Pseudomonas aeruginosa PAO1
Shewanella oneidensis MR-1
Vibrio cholerae O1 biovar El Tor str. N16961

**Gamma-Proteobacteria**
Acinetobacter baumannii AB0057
Acinetobacter calcoaceticus PHEA-2
Acinetobacter oleivorans DR1
Aeromonas hydrophila subsp. hydrophila ATCC 7966
Aeromonas salmonicida subsp. salmonicida A449
Aeromonas veronii B565
Alcanivorax borkumensis SK2
Alcanivorax dieselolei B5
Aliivibrio salmonicida LFI1238
Alkalilimnicola ehrlichii MLHE-1
Allochromatium vinosum DSM 180
Alteromonas macleodii str. 'Ionian Sea U7'
Alteromonas sp. SN2
Azotobacter vinelandii CA6
Cellvibrio japonicus Ueda107
Chromohalobacter salexigens DSM 3043
Citrobacter koseri ATCC BAA-895
Citrobacter rodentium ICC168
Colwellia psychrerythraea 34H
Cronobacter sakazakii ATCC BAA-894
Cronobacter turicensis z3032

**Gamma-Proteobacteria**
Dichelobacter nodosus VCS1703A
Dickeya dadantii Ech703
Dickeya zeae Ech1591
Edwardsiella ictaluri 93-146
Edwardsiella tarda C07-087
Enterobacter aerogenes KCTC 2190
Enterobacter asburiae LF7a
Enterobacter cloacae subsp. cloacae NCTC 9394
Enterobacter sp. 638
Enterobacteriaceae bacterium strain FGI 57
Erwinia amylovora ATCC 49946
Erwinia billingiae Eb661
Erwinia pyrifoliae Ep1/96
Erwinia sp. Ejp617
Erwinia tasmaniensis Et1/99
Escherichia coli O157:H7 str. EDL933
Escherichia fergusonii ATCC 35469
Ferrimonas balearica DSM 9799
Frateuria aurantia DSM 6220
Gammaproteobacteria gamma proteobacterium HdN
Glaciecola nitratireducens FR1064
Glaciecola psychrophila 170
Glaciecola sp. 4H-3-7+YE-5
Hahella chejuensis KCTC 2396
Halomonas elongata DSM 2581
Halorhodospira halophila SL1

**Gamma-Proteobacteria**
Halothiobacillus neapolitanus c2
Herminiimonas arsenicoxydans
Idiomarina loihiensis GSL 199
Kangiella koreensis DSM 16069
Legionella longbeachae NSW150
Listonella anguillarum M3
Marinobacter adhaerens HP15
Marinobacter aquaeolei VT8
Marinobacter hydrocarbonoclasticus ATCC 49840
Marinobacter sp. BSs20148
Marinomonas mediterranea MMB-1
Marinomonas posidonica IVIA-Po-181
Marinomonas sp. MWYL1
Methylococcus capsulatus str. Bath
Methylomonas methanica MC09
Methylophaga sp. JAM1
Morganella morganii subsp. morganii KT
Nitrosococcus halophilus Nc4
Nitrosococcus oceani ATCC 19707
Nitrosococcus watsonii C-113
Oceanimonas sp. GK1
Pantoea ananatis LMG 20103
Pantoea sp. At-9b
Pantoea vagans C9-1
Pectobacterium atrosepticum SCRI1043
Pectobacterium carotovorum subsp. carotovorum PCC21

**Gamma-Proteobacteria**
Pectobacterium sp. SCC3193
Pectobacterium wasabiae WPP163
Photobacterium profundum SS9
Photorhabdus asymbiotica
Photorhabdus luminescens subsp. laumondii TTO1
Proteus mirabilis BB2000
Providencia stuartii MRSN 2154
Pseudoalteromonas atlantica T6c
Pseudoalteromonas haloplanktis TAC125
Pseudoalteromonas sp. SM9913
Pseudomonas aeruginosa PA1
Pseudomonas brassicacearum subsp. brassicacearum NFM421
Pseudomonas denitrificans ATCC 13867
Pseudomonas entomophila L48
Pseudomonas fluorescens A506
Pseudomonas fulva 12-X
Pseudomonas mendocina ymp
Pseudomonas monteilii SB3101
*Pseudomonas poae RE*1-1-14*
Pseudomonas protegens Pf-5
Pseudomonas putida BIRD-1
Pseudomonas resinovorans NBRC 106553
Pseudomonas sp. TKP
Pseudomonas stutzeri DSM 4166
Pseudomonas syringae pv. syringae B728a
Pseudoxanthomonas spadix BD-a59

**Gamma-Proteobacteria**
Pseudoxanthomonas suwonensis 11-1
Psychrobacter arcticus 273-4
Psychrobacter cryohalolentis K5
Psychrobacter sp. PRwf-1
Psychromonas sp. CNPT3
Rahnella aquatilis CIP 78.65 = ATCC 33071
Rahnella sp. Y9602
Rhodanobacter sp. 2APBS1
Saccharophagus degradans 2-40
Salmonella bongori NCTC 12419
Salmonella enterica subsp. enterica serovar Typhi str. Ty21a
Serratia liquefaciens ATCC 27592
Serratia marcescens WW4
Serratia plymuthica AS9
Serratia proteamaculans 568
Serratia sp. AS12
Shewanella amazonensis SB2B
Shewanella baltica BA175
Shewanella denitrificans OS217
Shewanella frigidimarina NCIMB 400
Shewanella halifaxensis HAW-EB4
Shewanella loihica PV-4
Shewanella pealeana ATCC 700345
Shewanella piezotolerans WP3
Shewanella putrefaciens 200
Shewanella sediminis HAW-EB3

**Gamma-Proteobacteria**
Shewanella sp. ANA-3
Shewanella violacea DSS12
Shewanella woodyi ATCC 51908
Shigella boydii CDC 3083-94
Shigella flexneri 2a str. 301
Shigella sonnei Ss046
Simiduia agarivorans SA1 = DSM 21679
Stenotrophomonas maltophilia JV3
Teredinibacter turnerae T7901
Thalassolituus oleivorans MIL-1
Thioalkalivibrio sp. K90mix
Thioalkalivibrio sulfidophilus HL-EbGr7
Thiocystis violascens DSM 198
Thiomicrospira crunogena XCL-2
Vibrio alginolyticus NBRC 15630 = ATCC 17749
Vibrio anguillarum 775
Vibrio campbellii ATCC BAA-1116
Vibrio cholerae O395
Vibrio fischeri ES114
Vibrio furnissii NCTC 11218
Vibrio harveyi ATCC BAA-1116
Vibrio nigripulchritudo
Vibrio parahaemolyticus RIMD 2210633
Vibrio sp. EJY3
Vibrio splendidus LGP32
Vibrio vulnificus CMCP6

**Gamma-Proteobacteria**
Xanthomonas albilineans GPE PC73
Xanthomonas axonopodis pv. citrumelo F1
Xanthomonas campestris pv. vesicatoria str. 85-10
Xanthomonas citri subsp. citri Aw12879
Xanthomonas oryzae pv. oryzae KACC 10331
Xenorhabdus bovienii SS-2004
Xenorhabdus nematophila ATCC 19061
Xylella fastidiosa subsp. fastidiosa GB514
Yersinia enterocolitica subsp. enterocolitica 8081
Yersinia pestis Antiqua
Yersinia pseudotuberculosis YPIII

**Beta-Proteobacteria**
Achromobacter xylosoxidans NBRC 15126 = ATCC 27061
Acidithiobacillus caldus SM-1
Bordetella pertussis 18323
Candidatus Accumulibacter phosphatis clade IIA str. UW-1
Collimonas fungivorans Ter331
Gallionella capsiferriformans ES-2
Janthinobacterium sp. Marseille
Ralstonia pickettii 12J
Ralstonia solanacearum Po82
Variovorax paradoxus S110

Supplementary Table 11: Aer2-like pentapeptide tethers:

Organism identifier	locus	accession	pentapeptide
Al_mac_7736	I876_01970	YP_008194818.1	DWEAF
Al_mac_7736	I876_02010	YP_008194826.1	EWETF

Organism identifier	locus	accession	pentapeptide
Al_mac_7736	I876_02015	YP_008194827.1	EWESF
Al_sp._1413	ambt_16735	YP_004468654.1	EWEAF
Al_vin_90	Alvin_0183	YP_003442182.1	QWEEF
Al_vin_90	Alvin_1872	YP_003443828.1	
Al_vin_90	Alvin_2222	YP_003444173.1	
Al_vin_90	Alvin_2230	YP_003444181.1	
Gl_nit_1515	GNIT_1657	YP_004871766.1	EWKEF
Gl_sp._1395	Glaag_2576	YP_004434785.1	EWESF
Ha_che_746	HCH_00457	YP_431792.1	DWEVF
Ha_che_746	HCH_00458	YP_431793.1	DWEVF
Ha_hal_741	Hhal_2163	YP_001003729.1	EWEEF
Li_ang_7812	N175_16910	YP_008489689.1	EWEEF
Ma_med_1360	Marme_1102	YP_004312213.1	DWEEF
Ma_sp._859	Mmwyl1_3301	YP_001342141.1	GWEEF
Me_alc_1536	MEALZ_2872	YP_004918123.1	EWEEF
Me_met_1418	Metme_2154	YP_004513058.1	EWQDF
Ps_aer_479	PA0176	NP_248866.1	GWEEF
Ps_aer_7891	PA1S_gp3690	REF_DMTMMU:PA1S_gp3690	GWEEF
Ps_den_2356	H681_00805	YP_007655583.1	DWEEF
Ps_res_7713	PCA10_13880	YP_008101725.1	EWEEF
Ps_sp._1241	PSM_A2954	YP_004070018.1	EWEEF
Ps_suw_1301	Psesu_0059	YP_004145153.1	DWQEF
Ps_suw_1301	Psesu_1463	YP_004146541.1	
Ps_suw_1301	Psesu_1465	YP_004146543.1	EWAKF
Ps_suw_1301	Psesu_1466	YP_004146544.1	DWAEF
Sa_deg_468	Sde_3105	YP_528574.1	DWEDF
Sh_ama_634	Sama_3497	YP_929369.1	EWHEF
Sh_bal_241	Sbal175_2162	YP_006020732.1	EWEEF
Sh_loi_680	Shew_0111	YP_001092242.1	EWNEF

**Organism identifier**	**locus**	**accession**	**pentapeptide**
Sh_one_481	SO_2123	NP_717726.1	EWEEF
Sh_sed_917	Ssed_0184	YP_001471925.1	EWNEF
Sh_sp._679	Shewana3_2216	YP_869851.1	EWEDF
Sh_vio_130	SVI_0176	YP_003554925.1	EWNEF
Sh_woo_862	Swoo_0164	YP_001758560.1	EWNEF
Si_aga_2165	M5M_00415	YP_006915050.1	EWEEF
St_mal_1491	BurJV3_1158	YP_004791716.1	GWEEF
St_mal_1491	BurJV3_1903	YP_004792454.1	DWQEF
St_mal_1491	BurJV3_1904	YP_004792455.1	DWQEF
St_mal_1491	BurJV3_1908	YP_004792459.1	DWQEF
St_mal_1491	BurJV3_2459	YP_004793006.1	
St_mal_1491	BurJV3_3037	YP_004793581.1	
St_mal_1491	BurJV3_3580	YP_004794119.1	
St_mal_1491	BurJV3_3943	YP_004794481.1	
Te_tur_1125	TERTU_1341	YP_003072897.1	EWEDF
Te_tur_1125	TERTU_2935	YP_003074319.1	
Th_cru_598	Tcr_0553	YP_390823.1	DWSDF
Th_cru_598	Tcr_2004	YP_392268.1	
Th_ole_2361	TOL_2508	YP_007683144.1	EWEEF
Th_vio_1521	Thivi_0439	YP_006412631.1	QWEEF
Th_vio_1521	Thivi_1211	YP_006413359.1	DWEEF
Th_vio_1521	Thivi_1222	YP_006413370.1	EWSEF
Vi_ang_1433	VAA_01905	YP_004577835.1	EWEEF
Vi_cho_1795	VC395_0082	YP_002818346.1	EWESF
Vi_cho_1795	VC395_A1113	YP_002822179.1	EWEEF
Vi_cho_319	VC0098	NP_229757.1	EWESF
Vi_cho_319	VCA1092	NP_233472.1	EWEEF
Vi_fur_1564	vfu_B00980	YP_005049501.1	EWEEF
Vi_nig_7850	VIBNI_B0011	YP_008640924.1	EWEEF

Organism identifier	locus	accession	pentapeptide
Vi_nig_7850	VIBNI_B0830	YP_008641677.1	
Vi_vul_1326	VV2_1165	NP_763073.1	EWEEF
Xa_alb_65	XALc_0649	YP_003375155.1	DWEEF
Xa_alb_65	XALc_1357	YP_003375852.1	
Xa_alb_65	XALc_1361	YP_003375856.1	QWRDF
Xa_alb_65	XALc_1362	YP_003375857.1	HWHEF
Xa_alb_65	XALc_1364	YP_003375859.1	QWQEF
Xa_alb_65	XALc_1365	YP_003375860.1	SWQEF
Xa_alb_65	XALc_1926	YP_003376405.1	
Xa_alb_65	XALc_2151	YP_003376626.1	NWQEF
Xa_alb_65	XALc_2152	YP_003376627.1	DWQEF
Xa_alb_65	XALc_2153	YP_003376628.1	DWQEF
Xa_alb_65	XALc_3131	YP_003377604.1	
Xa_axo_1502	XACM_0614	YP_004850217.1	
Xa_axo_1502	XACM_1288	YP_004850870.1	DWQDF
Xa_axo_1502	XACM_1685	YP_004851263.1	
Xa_axo_1502	XACM_1913	YP_004851485.1	NWQEF
Xa_axo_1502	XACM_1918	YP_004851490.1	
Xa_axo_1502	XACM_1920	YP_004851492.1	NWQEF
Xa_axo_1502	XACM_1921	YP_004851493.1	DWQEF
Xa_axo_1502	XACM_1922	YP_004851494.1	
Xa_axo_1502	XACM_1923	YP_004851495.1	SWQEF
Xa_axo_1502	XACM_1925	YP_004851496.1	NWAEF
Xa_axo_1502	XACM_1926	YP_004851497.1	DWSEF
Xa_axo_1502	XACM_1927	YP_004851498.1	QWQDF
Xa_axo_1502	XACM_1929	YP_004851500.1	QWQDF
Xa_axo_1502	XACM_1930	YP_004851501.1	
Xa_axo_1502	XACM_1932	YP_004851503.1	NWQEF
Xa_axo_1502	XACM_1933	YP_004851504.1	SWQEF

Organism identifier	locus	accession	pentapeptide
Xa_axo_1502	XACM_3051	YP_004852602.1	
Xa_cam_666	XCV0669	YP_362400.1	
Xa_cam_666	XCV1702	YP_363433.1	
Xa_cam_666	XCV1933	YP_363664.1	NWQEF
Xa_cam_666	XCV1938	YP_363669.1	
Xa_cam_666	XCV1939	YP_363670.1	NWQEF
Xa_cam_666	XCV1940	YP_363671.1	DWQEF
Xa_cam_666	XCV1941	YP_363672.1	
Xa_cam_666	XCV1942	YP_363673.1	SWQEF
Xa_cam_666	XCV1944	YP_363675.1	SWQEF
Xa_cam_666	XCV1945	YP_363676.1	NWAEF
Xa_cam_666	XCV1947	YP_363678.1	DWSEF
Xa_cam_666	XCV1948	YP_363679.1	QWQDF
Xa_cam_666	XCV1951	YP_363682.1	QWQDF
Xa_cam_666	XCV1952	YP_363683.1	
Xa_cam_666	XCV1954	YP_363685.1	NWQEF
Xa_cam_666	XCV1955	YP_363686.1	SWQEF
Xa_cam_666	XCV3261	YP_364992.1	
Xa_cit_2353	XCAW_02407	YP_007650389.1	
Xa_cit_2353	XCAW_02490	YP_007650471.1	DWQEF
Xa_cit_2353	XCAW_02492	YP_007650473.1	
Xa_cit_2353	XCAW_02493	YP_007650474.1	QWQDF
Xa_cit_2353	XCAW_02495	YP_007650476.1	QWQDF
Xa_cit_2353	XCAW_02496	YP_007650477.1	DWSEF
Xa_cit_2353	XCAW_02497	YP_007650478.1	NWAEF
Xa_cit_2353	XCAW_02498	YP_007650479.1	SWQEF
Xa_cit_2353	XCAW_02499	YP_007650480.1	SWQEF
Xa_cit_2353	XCAW_02500	YP_007650481.1	
Xa_cit_2353	XCAW_02501	YP_007650482.1	NWQEF

Organism identifier	locus	accession	pentapeptide
Xa_cit_2353	XCAW_02502	YP_007650483.1	NWQEF
Xa_cit_2353	XCAW_02504	YP_007650485.1	
Xa_cit_2353	XCAW_02508	YP_007650489.1	NWQEF
Xa_cit_2353	XCAW_03417	YP_007651390.1	
Xa_cit_2353	XCAW_03970	YP_007651933.1	
Xa_cit_2353	XCAW_04466	YP_007652428.1	
Xa_ory_584	XOO2840	YP_201479.1	DWAEF
Xa_ory_584	XOO2842	YP_201481.6	NWAEF
Xa_ory_584	XOO2844	YP_201483.1	SWQEF
Xa_ory_584	XOO2845	YP_201484.1	
Xa_ory_584	XOO2847	YP_201486.1	NWQDF
Xa_ory_584	XOO2848	YP_201487.6	DWQDF

Supplementary Table 12: McpA-like C-terminal motif

Organism identifier	locus	accession	pentapeptide
Al_mac_7736	I876_01990	YP_008194822.1	DIELF-
Al_mac_7736	I876_01950	YP_008194814.1	EVELF-
Al_sp._1413	ambt_16755	YP_004468658.1	EVELF-
Gl_sp._1395	Glaag_2582	YP_004434791.1	DLELF-
Ma_med_1360	Marme_1096	YP_004312207.1	EIDLF-
Ma_sp._859	Mmwyl1_3295	YP_001342135.1	DIDLF-
Sh_sp._679	Shewana3_2222	YP_869857.1	EIELF-
Sh_one_481	SO_2117	NP_717720.2	EIELF-
Sh_bal_241	Sbal175_2156	YP_006020726.1	EIELF-
Ps_den_2356	H681_00825	YP_007655587.1	EVELF-
Ps_res_7713	PCA10_13920	YP_008101729.1	EVELF-
Ps_aer_479	PA0180	NP_248870.1	EVELF-
Ps_aer_7891	PA1S_gp3694	REF_DMTMMU:PA1S_gp3694	EVELF-

**Organism identifier**	**locus**	**accession**	**pentapeptide**
Th_cru_598	Tcr_0759	YP_391029.1	EIDLF-
Ha_hal_741	Hhal_2159	YP_001003725.1	DVELF-
Gl_nit_1515	GNIT_1661	YP_004871770.1	DIELF-
Me_alc_1536	MEALZ_2878	YP_004918129.1	DIELF-
Me_met_1418	Metme_2161	YP_004513065.1	DVELF-
Fr_aur_1557	Fraau_2042	YP_005378108.1	DIDLF-
Rh_sp._1523	R2APBS1_2961	YP_007591271.1	EIELF-
Al_vin_90	Alvin_2234	YP_003444185.1	DIELF-
Th_vio_1521	Thivi_1219	YP_006413367.1	DIELF-
Ha_che_746	HCH_00449	YP_431784.1	DIELF-
Sa_deg_468	Sde_3111	YP_528580.1	EIVLY-
Te_tur_1125	TERTU_1204	YP_003072781.1	DIELYE
Ps_suw_1301	Psesu_1530	YP_004146608.1	TVELF-
Xa_alb_65	XALc_1440	YP_003375935.1	TVELF-
St_mal_1491	BurJV3_1974	YP_004792525.1	TVELF-
Xa_ory_584	XOO2558	YP_201197.1	TVELF-
Xa_cit_2353	XCAW_01830	YP_007649819.1	TVELF-
Xa_axo_1502	XACM_2022	YP_004851592.1	TVELF-
Xa_cam_666	XCV2044	YP_363775.1	TVELF-
Vi_vul_1326	VV2_1160	NP_763069.1	EVELF-
Vi_nig_7850	VIBNI_B0016	YP_008640928.1	EVELF-
Vi_fur_1564	vfu_B00976	YP_005049497.1	EVELF-
Vi_cho_1795	VC395_A1109	YP_002822175.1	EVELF-
Vi_cho_319	VCA1088	NP_233469.1	EVELF-
Vi_ang_1433	VAA_01909	YP_004577831.1	EVELF-
Li_ang_7812	N175_16890	YP_008489685.1	EVELF-

Supplementary Note 1


```
{
  "patterns": [
    {
      "npos": [
        {
          "count": "{0}"
          "name": "TM",
          "resource": "tmhmm2",
        }
      ],
      "pos": [
        {
          "name": "^",
          "resource": "regarch"
        },
        {
          "name": "Response_reg",
          "resource": "pfam30"
        },
        {
          "name": "$",
          "resource": "regarch"
        }
      ]
    }
  ]
}
```


Supplementary References

1. Rosario, M. M. L. & Ordal, G. W. CheC and CheD interact to regulate methylation of Bacillus subtilis methyl-accepting chemotaxis proteins. *Mol. Microbiol.* **21**, 511–518 (1996).
2. Glekas, G. D. *et al.* Elucidation of the multiple roles of CheD in Bacillus subtilis chemotaxis. *Mol. Microbiol.* **86**, 743–756 (2012).
3. Huang, Z. *et al.* Cross Talk between Chemosensory Pathways That Modulate Chemotaxis and Biofilm Formation. *mBio* **10**, (2019).
4. Ortega, D. R. *et al.* Assigning chemoreceptors to chemosensory pathways in Pseudomonas aeruginosa. *Proc. Natl. Acad. Sci. U. S. A.* (2017) doi:10.1073/pnas.1708842114.

5. Güvener, Z. T., Tifrea, D. F. & Harwood, C. S. Two different Pseudomonas aeruginosa chemosensory signal transduction complexes localize to cell poles and form and remould in stationary phase. *Mol. Microbiol.* **61**, 106–118 (2006).

6. Herrera Seitz, M. K., Frank, V., Massazza, D. A., Vaknin, A. & Studdert, C. A. Bacterial chemoreceptors of different length classes signal independently. *Mol Microbiol* **93**, 814–22 (2014).

7. Kim, K. K., Yokota, H. & Kim, S. H. Four-helical-bundle structure of the cytoplasmic domain of a serine chemotaxis receptor. *Nature* **400**, 787–92 (1999).

8. Li, M. & Hazelbauer, G. L. Cellular Stoichiometry of the Components of the Chemotaxis Signaling Complex. *J Bacteriol* **186**, 3687–3694 (2004).

9. Cannistraro, V. J., Glekas, G. D., Rao, C. V. & Ordal, G. W. Cellular stoichiometry of the chemotaxis proteins in Bacillus subtilis. *J. Bacteriol.* **193**, 3220–3227 (2011).

10. Briegel, A. *et al.* New Insights into Bacterial Chemoreceptor Array Structure and Assembly from Electron Cryotomography. *Biochemistry* **53**, 1575–1585 (2014).

11. Masduki, A. *et al.* Isolation and characterization of chemotaxis mutants and genes of Pseudomonas aeruginosa. *J Bacteriol* **177**, 5 (1995).

12. Gosink, K. K., Kobayashi, R., Kawagishi, I. & Hase, C. C. Analyses of the roles of the three cheA homologs in chemotaxis of Vibrio cholerae. *J. Bacteriol.* **184**, 1767–71 (2002).

13. Li, J., Romine, M. F. & Ward, M. J. Identification and analysis of a highly conserved chemotaxis gene cluster in Shewanella species. *FEMS Microbiol Lett* **273**, 180–6 (2007).

14. Ringgaard, S., Hubbard, T., Mandlik, A., Davis, B. M. & Waldor, M. K. RpoS and quorum sensing control expression and polar localization of Vibrio cholerae chemotaxis cluster III proteins in vitro and in vivo. *Mol. Microbiol.* **97**, 660–675 (2015).

15. Schuster, M., Hawkins, A. C., Harwood, C. S. & Greenberg, E. P. The Pseudomonas aeruginosa RpoS regulon and its relationship to quorum sensing. *Mol. Microbiol.* **51**, 973–985 (2004).

16. Harris, H. W., El-Naggar, M. Y. & Nealson, K. H. Shewanella oneidensis MR-1 chemotaxis proteins and electron-transport chain components essential for congregation near insoluble electron acceptors. *Biochem Soc Trans* **40**, 1167–77 (2012).

17. Khmelenina, V. N., Kalyuzhnaya, M. G., Starostina, N. G., Suzina, N. E. & Trotsenko, Y. A. Isolation and Characterization of Halotolerant Alkaliphilic Methanotrophic Bacteria from Tuva Soda Lakes. *Curr. Microbiol.* **35**, 257–261 (1997).

18. Greer-Phillips, S. E. *et al.* The Aer2 receptor from Vibrio cholerae is a dual PAS-heme oxygen sensor. *Mol. Microbiol.* **109**, 209–224 (2018).

19. Garcia, D., Orillard, E., Johnson, M. S. & Watts, K. J. Gas Sensing and Signaling in the PAS-Heme Domain of the Pseudomonas aeruginosa Aer2 Receptor. *J. Bacteriol.* **199**, e00003-17 (2017).

20. Kim, H. E. *et al.* Identification and characterization of the chemotactic transducer in Pseudomonas aeruginosa PAO1 for positive chemotaxis to trichloroethylene. *J Bacteriol* **188**, 6700–2 (2006).

21. Ferris, H. U., Zeth, K., Hulko, M., Dunin-Horkawicz, S. & Lupas, A. N. Axial helix rotation as a mechanism for signal regulation inferred from the crystallographic analysis of the E. coli serine chemoreceptor. *J. Struct. Biol.* **186**, 349–356 (2014).

22. Airola, M. V. *et al.* Architecture of the Soluble Receptor Aer2 Indicates an In-Line Mechanism for PAS and HAMP Domain Signaling. *J. Mol. Biol.* **425**, 886–901 (2013).

23. Sawai, H. *et al.* Structural basis for oxygen sensing and signal transduction of the heme-based sensor protein Aer2 from Pseudomonas aeruginosa. *Chem. Commun.* **48**, 6523–6525 (2012).

24. Airola, M. V. *et al.* HAMP domain conformers that propagate opposite signals in bacterial chemoreceptors. *PLoS Biol* **11**, e1001479 (2013).
