# Supplementary material for: Repurposing a chemosensory macromolecular machine
Source: Nat Commun. 2020 Apr 27;11:2041. doi: 10.1038/s41467-020-15736-5 (PMC7184735; doi:10.1038/s41467-020-15736-5)
Supplement: Supplementary file 3 — Description of Additional Supplementary Information [file 41467_2020_15736_MOESM3_ESM.pdf]

Description of Additional Supplementary Files

File Name: Supplementary Data 1
Description: Files used to produce the homology models as described in Supplementary Table 8.

File Name: Supplementary Data 2
Description: Phylogenetic trees in Figure 4 and Supplementary Figure 1, 2 and 4.
