# Supplementary material for: Repurposing a chemosensory macromolecular machine
Source: Nat Commun. 2020 Apr 27;11:2041. doi: 10.1038/s41467-020-15736-5 (PMC7184735; doi:10.1038/s41467-020-15736-5)
Supplement: Supplementary file 6 — Reporting Summary [file 41467_2020_15736_MOESM6_ESM.pdf]

nature research

Corresponding author(s): Grant Jensen, Ariane Briegel

Last updated by author(s): Mar 10, 2020

Reporting Summary

Nature Research wishes to improve the reproducibility of the work that we publish. This form provides structure for consistency and transparency in reporting. For further information on Nature Research policies, see Authors & Referees and the Editorial Policy Checklist .

Statistics

For all statistical analyses, confirm that the following items are present in the figure legend, table legend, main text, or Methods section.

n/a	Confirmed	
☐	☒	The exact sample size (*n*) for each experimental group/condition, given as a discrete number and unit of measurement
☐	☒	A statement on whether measurements were taken from distinct samples or whether the same sample was measured repeatedly
☒	☐	The statistical test(s) used AND whether they are one- or two-sided<br>*Only common tests should be described solely by name; describe more complex techniques in the Methods section.*
☒	☐	A description of all covariates tested
☒	☐	A description of any assumptions or corrections, such as tests of normality and adjustment for multiple comparisons
☒	☐	A full description of the statistical parameters including central tendency (e.g. means) or other basic estimates (e.g. regression coefficient) AND variation (e.g. standard deviation) or associated estimates of uncertainty (e.g. confidence intervals)
☒	☐	For null hypothesis testing, the test statistic (e.g. *F*, *t*, *r*) with confidence intervals, effect sizes, degrees of freedom and *P* value noted<br>*Give P values as exact values whenever suitable.*
☒	☐	For Bayesian analysis, information on the choice of priors and Markov chain Monte Carlo settings
☒	☐	For hierarchical and complex designs, identification of the appropriate level for tests and full reporting of outcomes
☒	☐	Estimates of effect sizes (e.g. Cohen's *d*, Pearson's *r*), indicating how they were calculated

Our web collection on statistics for biologists contains articles on many of the points above.

Software and code

Policy information about availability of computer code

Data collection	UCSFTomo - Acquisition of individual tilt series in the electron microscope.
Data analysis	CD-HIT v4.6 - fast clustering of protein sequences by similarity<br>MAFFT v7.305b - Multiple Sequence Alignment (MSA)<br>Gblocks v0.91b - Eliminate poorly aligned columns in MSA<br>VMD v1.9.3 - Visualization of atomic models.<br>MODELLER v9.17 - Used to build homology models<br>Jalview v2.10.1 - Visualization of MSA<br>JNet4 - Prediction of secondary structures from protein sequences<br>BLAST v2.7.1+ - Protein similarity search tool<br>HMMER v3.1b2 - Hidden Markov Model search tool for protein sequences and domain models.<br>RAxML v8.2.10 - Phylogenetic reconstruction<br>TreeCollapseCL4 v3.0 - Collapse nodes with poorly supported nodes in phylogenetic tree<br>WebLogo v3.6.0 - Build Sequence Logos<br>IMOD - Electron Tomography visualization and data processing<br>Dynamo - Subtomogram averages<br>RegArch 1.1 - https://www.npmjs.com/package/regarch - Find protein architectures that match a protein architecture pattern<br>sideview-profile-average - https://www.npmjs.com/package/sideview-profile-average - Calculate 1D average electron density profiles across a path in tomograms<br>https://beta.observablehq.com/@daviortega/generic-notebook-to-analyse-1d-averaged-electron-density-p - visualize the 1D electron density profiles calculated by sideview-profile-average

For manuscripts utilizing custom algorithms or software that are central to the research but not yet described in published literature, software must be made available to editors/reviewers. We strongly encourage code deposition in a community repository (e.g. GitHub). See the Nature Research guidelines for submitting code & software for further information.

Data

Policy information about availability of data

All manuscripts must include a data availability statement. This statement should provide the following information, where applicable:

- Accession codes, unique identifiers, or web links for publicly available datasets
- A list of figures that have associated raw data
- A description of any restrictions on data availability

Tomograms are available in the Electron Tomography Database – Caltech at https://etdb.caltech.edu and their identifiers are listed in the Supplementary Table 6. Phylogenetic trees in Fig. 4, Supplementary Figure 1, 2 and 4 are available in Supplementary Data 1. The homology models in Fig. 2D are available in Supplementary Data 2. Other data are available from the corresponding authors upon reasonable request.

Field-specific reporting

Please select the one below that is the best fit for your research. If you are not sure, read the appropriate sections before making your selection.

☒ Life sciences ☐ Behavioural & social sciences ☐ Ecological, evolutionary & environmental sciences

For a reference copy of the document with all sections, see nature.com/documents/nr-reporting-summary-flat.pdf

Life sciences study design

All studies must disclose on these points even when the disclosure is negative.

Sample size	The number of species in the bioinformatics analysis was taken as a randomly chosen non-redundant set present in the MiST database in 2017. The set contains a representative of all major genus of gamma-proteobacteria. The number of chemotaxis systems analised were the result of many filtering steps described in the methods section but mainly a non-redundant set of all CheAs present in Proteobacteria as present in MiST database in 2017. The final number the CheA in the study covers the sequence diversity of all CheA in Proteobacteria at 85% identity.
Data exclusions	Chemotaxis F7 systems were excluded from the analysis if did not contained one of the 3 marker (CheA, CheB or CheR) in the gene cluster. Systems with more than one CheB or CheR were also excluded. This criteria was used previously in Wuichet and Zhulin 2010.
Replication	Multiple inferences were calculated for Phylogenetic tree to find the tree with best score of maximum likelihood. Bootstrap analysis was performed with over 500 replicates. All replicates were successful.<br><br>Multiple tomograms were taken from each mutant or growth condition when applicable.
Randomization	This is not relevant to our study. We selected all organisms (4) with predicted F7 systems and with tomograms available in the Jensen Lab internal database. Two of these organisms have been extensively studied and were chosen for further analysis (mutagenesis) for being model organisms.
Blinding	Chemotaxis systems were blindly classified and grouped based on phylogenetics and gene cluster arrangement only.

Reporting for specific materials, systems and methods

We require information from authors about some types of materials, experimental systems and methods used in many studies. Here, indicate whether each material, system or method listed is relevant to your study. If you are not sure if a list item applies to your research, read the appropriate section before selecting a response.

Materials & experimental systems

n/a	Involved in the study	
☒	☐	Antibodies
☒	☐	Eukaryotic cell lines
☒	☐	Palaeontology
☒	☐	Animals and other organisms
☒	☐	Human research participants
☒	☐	Clinical data

Methods

n/a	Involved in the study	
☒	☐	ChIP-seq
☒	☐	Flow cytometry
☒	☐	MRI-based neuroimaging
